# Engineering *Pseudomonas taiwanensis* VLB120 for regio- and stereospecific hydroxylation of l-lysine fueled by the Weimberg pathway

**DOI:** 10.1186/s12934-026-02931-0

**Published:** 2026-02-19

**Authors:** Philipp Nerke, Julian Handke, Georg Hubmann, Stephan Lütz

**Affiliations:** Chair for Bioprocess Engineering, Department of Biochemical and Chemical Engineering, TU Dortmund University, Emil-Figge-Straße 66, 44227 Dortmund, Germany

**Keywords:** Lysine hydroxylation, Hydroxy-l-lysine, α-ketoglutarate-dependent oxygenase, Lysine hydroxylase, *Pseudomonas taiwanensis* VLB120, Weimberg pathway

## Abstract

**Background:**

Hydroxy-l-lysines are versatile chiral building blocks and can be obtained by hydroxylation of the amino acid l-lysine. The conversion is catalyzed by α-ketoglutarate-dependent lysine dioxygenases (KDOs), which belong to the superfamily of Fe^2+^/α-ketoglutarate-dependent oxygenases. These enzymes are highly regio- and stereoselective; however, they require α-ketoglutarate (α-KG) as a cosubstrate. Apart from the costly direct addition of α-KG, it can be generated via cellular metabolism from inexpensive and renewable carbon sources, such as d-xylose. Therefore, we engineered a *Pseudomonas taiwanensis* VLB120 chassis to efficiently convert l-lysine to hydroxy-l-lysine using KDOs with the supply of α-KG from d-xylose as the sole carbon source via the Weimberg pathway.

**Results:**

For the generation of a suitable whole-cell biocatalyst, we investigated the l-lysine catabolism of *P. taiwanensis* VLB120 and created a mutant strain that is deficient in l-lysine catabolism to minimize l-lysine degradation and to facilitate complete conversion via the biotransformation reaction. Next, a library of KDO genes was heterologously expressed in the engineered chassis strain *P. taiwanensis* VLB120∆C∆3. The hydroxylation of l-lysine was assessed in biotransformations with growing cells and d-xylose to supply α-KG via the Weimberg pathway. Hydroxy-l-lysine was successfully produced by strains harboring KDOs that hydroxylate the C-4 position of l-lysine. We further explored the three most promising whole-cell biocatalysts and investigated the influence of increased concentrations of the substrate l-lysine and the metal cofactor Fe^2+^. Finally, the engineered strain expressing a KDO from *Flavobacterium* species was grown in stirred-tank bioreactors and was able to produce 8.7 ± 0.3 g L^−1^ hydroxy-l-lysine with a space-time yield of 98.6 ± 3.4 mg L h^−1^ and a specific product yield on biocatalyst (Y_Hyl/X_) of 1.68 ± 0.07 g g_CDW_^−1^. The supply of α-KG via the Weimberg pathway proved very efficient, as approximately every second molecule of d-xylose which was converted and entered the central carbon metabolism was used for the biotransformation reaction (Y_Hyl/Xyl,net_ = 0.48 ± 0.02 mol mol^−1^).

**Conclusions:**

We successfully established a whole-cell biocatalyst for the synthesis of hydroxy-l-lysine from l-lysine and d-xylose and demonstrated multigram-scale production with our engineered strain. Our work lays the foundation for whole-cell bioprocesses utilizing Fe^2+^/α-ketoglutarate-dependent oxygenases fueled by the Weimberg pathway.

**Supplementary Information:**

The online version contains supplementary material available at 10.1186/s12934-026-02931-0.

## Background

Hydroxy-l-lysines represent building blocks for active pharmaceutical ingredients (APIs) such as the HIV protease inhibitor palinavir [1] and potential APIs against cancer, such as tambromycin [2], the proteasome inhibitors cepafungin I and glidobactin A [3, 4], and the protein kinase inhibitor (−)-balanol [5]. Moreover, decarboxylation of hydroxy-l-lysines provides access to chiral amino alcohols, which can be used as chiral auxiliaries and building blocks for specialized bio-based polymers [6–8].The chemical synthesis of enantiopure hydroxy-l-lysines is complex, requiring costly reagents and many reaction steps, which results in low overall yields [1, 9]. In 2014, Fe^2+^/α-ketoglutarate-dependent oxygenases were discovered, which catalyze the regio- and stereoselective hydroxylation of l-lysine [10]. This enzyme superfamily is of great interest for industrial applications due to its chemical versatility and high selectivity [11]. With the lysine hydroxylases, termed KDOs, specific hydroxy-l-lysine isomers can be synthesized from the inexpensive and readily available amino acid l-lysine. Depending on the specific enzyme, KDOs provide access to (3*S*)-3-hydroxy-l-lysine, (4*R*)-4-hydroxy-l-lysine, or (4*S*)-4-hydroxy-l-lysine [4, 10]. Recently, additional KDOs were discovered, which catalyze the synthesis of (5*R*)-5-hydroxy-l-lysine and (5*S*)-5-hydroxy-l-lysine from free-standing l-lysine [12]. However, the application of KDOs suffers from limited enzyme stabilities and low activities [7]. Enzyme immobilization has been shown to significantly enhance the stability of the enzymes, resulting in increased productivity [7]. In addition to the substrate l-lysine, the enzymes require stoichiometric quantities of the expensive cosubstrate α-ketoglutarate (α-KG), which is converted to succinate and CO_2_ during the reaction.

To circumvent the direct addition of α-KG, it can be supplied from less expensive substrates, for example, by utilizing enzymatic cascades [13] or via cellular metabolism [14]. However, the intracellular availability of α-KG is strongly dependent on the utilized metabolic pathway(s). Therefore, elaborate metabolic engineering strategies have been employed to increase the availability of α-KG in cell factories utilizing Fe^2+^/α-ketoglutarate-dependent oxygenases, for example, by enhancing the expression of tricarboxylic acid (TCA) cycle genes [15], knockout of α-ketoglutarate dehydrogenase [16–18], or dynamic control of α-ketoglutarate dehydrogenase [19, 20]. While most studies aiming to increase the availability of α-KG were performed using glucose as a substrate, the Weimberg pathway, an oxidative xylose pathway, represents a promising alternative for use with Fe^2+^/α-ketoglutarate-dependent oxygenases. The pentose d-xylose is the most abundant monosaccharide building block in lignocellulosic biomass after d-glucose and thus represents an attractive second-generation renewable feedstock for use in bioprocesses [21]. In the Weimberg pathway, d-xylose is converted in five steps to α-KG, which subsequently enters the central carbon metabolism. Due to the short and unbranched pathway, the Weimberg pathway is ideally suited to supply α-KG as a cosubstrate for α-ketoglutarate-dependent biotransformations. The Weimberg pathway was originally discovered in *Pseudomonas* species [22], which have emerged as efficient and versatile production hosts [23–25]. A *Pseudomonas* strain that natively catabolizes d-xylose via the Weimberg pathway is *Pseudomonas taiwanensis* VLB120 [26]. The solvent-tolerant strain has been efficiently utilized for oxygenase-based biocatalysis [27–29] and represents a promising *Pseudomonas* chassis for bioprocesses [23, 30]. Moreover, the Weimberg pathway in *P. taiwanensis* VLB120 has been characterized in detail in previous studies [26, 31]. The uptake of d-xylose into the periplasm is presumably mediated by the outer membrane porin OprB. Within the periplasm, the pyrroloquinoline quinone (PQQ)-dependent glucose dehydrogenase Gcd (PVLB_05240) catalyzes the oxidation of d-xylose to d-xylonolactone. This reaction concurrently links the Weimberg pathway to aerobic respiration through the generation of ubiquinol. d-Xylonolactone is subsequently hydrolyzed to d-xylonate, either spontaneously or via the action of a xylonolactonase encoded by PVLB_05820 and PVLB_12345. Transport of d-xylonate across the cytoplasmic membrane is facilitated by the transporters encoded by *gntP* and PVLB_18545 [31]. In the cytoplasm, xylonate dehydratase (PVLB_18565) catalyzes the conversion of d-xylonate to 2-keto-3-deoxy-d-xylonate, which is further dehydrated by 2-keto-3-deoxy-d-xylonate dehydratase (PVLB_18560) to yield α-ketoglutaric semialdehyde. Finally, α-ketoglutaric semialdehyde is oxidized by α-ketoglutaric semialdehyde dehydrogenase (PVLB_11380, PVLB_18510, PVLB_18550) to α-KG, which feeds into the central carbon metabolism.

One challenge in using pseudomonads for the hydroxylation of l-lysine is that they possess multiple interconnected pathways for catabolism of l-lysine, which are divided into the 5-aminovalerate branch and the 2-aminoadipate branch [32, 33]. In the 5-aminovalerate branch, l-lysine is converted to 5-aminopentanamide and subsequently 5-aminovalerate by the action of lysine 2-monooxygenase (DavB) and 5-aminopentanamidase (DavA). Another pathway proceeds via lysine decarboxylase (LdcC/LdcA), yielding 1,5-diaminopentane (cadaverine), which is subsequently converted to 1-piperideine and 5-aminovalerate by the action of cadaverine aminotransferase and 1-piperideine dehydrogenase. In the 2-aminoadipate branch, l-lysine is converted into d-lysine via a racemase (Alr) and subsequently into ∆^1^-piperideine-2-carboxylate, l-pipecolate, ∆^1^-piperideine-6-carboxylate, and 2-aminoadipate by the enzymes AmaD, DpkA, AmaA, and AmaB [34, 35]. Additionally, l-lysine can be converted by an aminotransferase (AruH), yielding α-keto-ε-aminohexanoate, which spontaneously reacts to ∆^1^-piperideine-2-carboxylate and is then further converted into 2-aminoadipate [36]. It is likely that catabolism of hydroxy-l-lysine proceeds via enzymes from l-lysine catabolism, as it has been shown that 5-hydroxy-l-lysine is converted via the monooxygenase pathway and the racemase pathway in *Pseudomonas fluorescens* [37]. While best studied in *Pseudomonas putida* and *Pseudomonas aeruginosa*, the catabolic pathways of l-lysine and hydroxy-l-lysine have not yet been investigated in *Pseudomonas taiwanensis* VLB120.

**Fig. 1:**
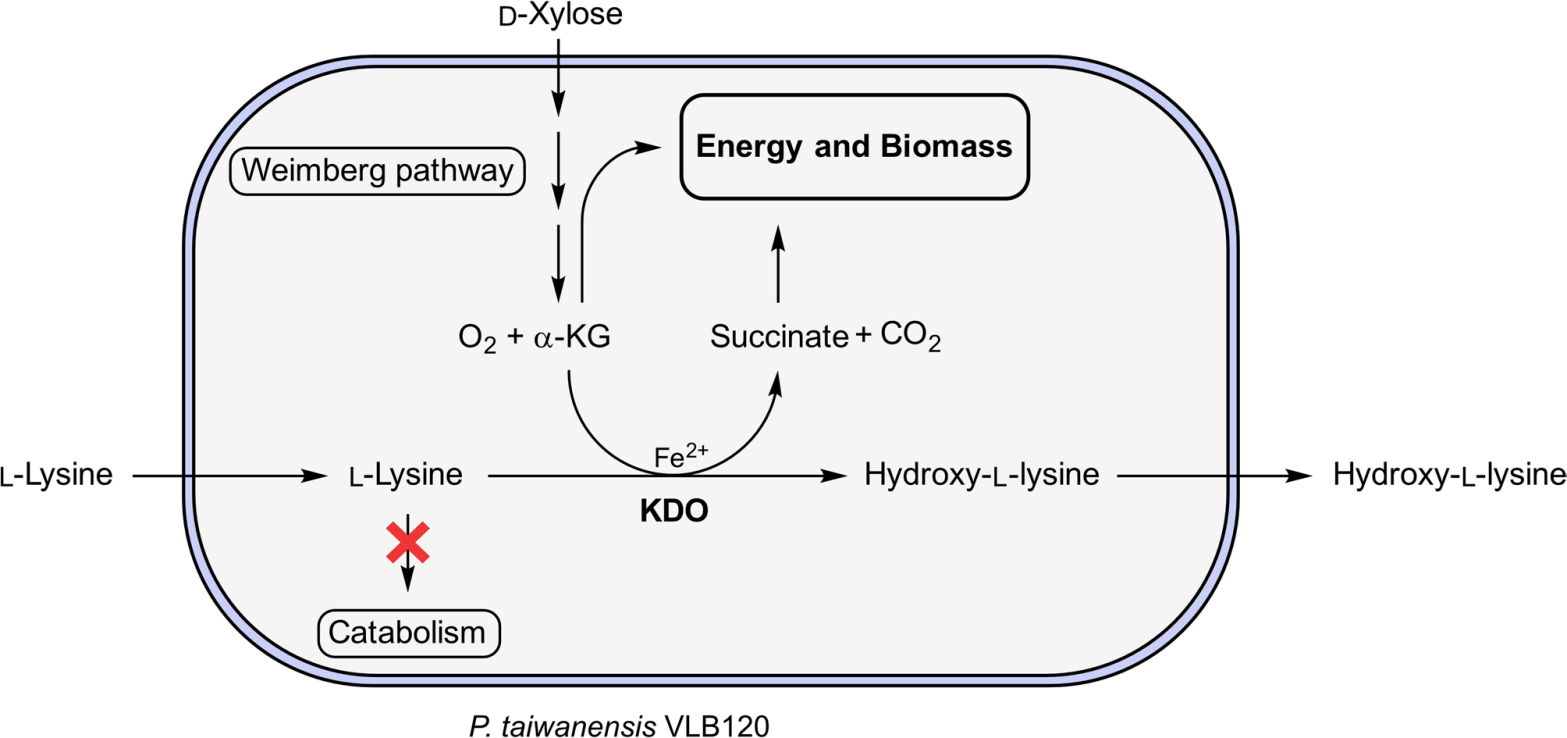
Schematic presentation of the whole-cell biocatalyst. The renewable carbon source d-xylose is taken up and converted into α-ketoglutarate (α-KG) via the Weimberg pathway. α-KG is converted via the endogenous metabolism or by the introduced α-ketoglutarate-dependent lysine dioxygenase (KDO). To minimize l-lysine degradation and to facilitate complete conversion via the KDO reaction, l-lysine catabolic pathways are deactivated by suitable gene knockouts. Transport of l-lysine and hydroxy-l-lysine across the bacterial cell membrane is performed by endogenous transporters, most likely ABC-type uptake systems for import and LysE-type efflux systems for export, as observed in other pseudomonads [38, 39]

In this study, we present a novel biosynthetic route for the hydroxylation of l-lysine employing *P. taiwanensis* VLB120 as a whole-cell biocatalyst with d-xylose as the sole carbon and energy source (Fig. 1). For that, we investigated the strains’ ability to catabolize l-lysine and knocked out the genes PVLB_23330, PVLB_08625, and PVLB_11490, which encode putative homologs of lysine 2-monooxygenase, lysine decarboxylase, and an aminotransferase, respectively. This eliminated the ability of *P. taiwanensis* VLB120 to grow on l-lysine as the sole carbon and energy source. We then expressed a library of twelve KDO-encoding genes in the engineered chassis strain and performed biotransformations with growing cells. Product formation was only detectable for the strains with KDOs catalyzing the hydroxylation of the C-4 position, but not the C-3 position. We further tested the three best-performing strains and selected the strain *P. taiwanensis* VLB120∆C∆3 pCom10lac_*Fspe*KDO for closer characterization. Finally, we successfully transferred the whole-cell biotransformation to stirred-tank bioreactors, demonstrating gram-scale production of (4*R*)-4-hydroxy-l-lysine from l-lysine and d-xylose.

##  Materials and methods

### Chemicals and culture media

Chemicals were purchased from Carl Roth GmbH + Co. KG (Karlsruhe, Germany), Sigma-Aldrich Chemie AG (St. Louis, USA), and Merck KGaA (Darmstadt, Germany). d-Xylose and l-lysine were obtained from Sigma-Aldrich. d-Lysine hydrochloride was purchased from Carl Roth. PCR primers were purchased from Sigma-Aldrich.

Bacterial cultures were cultivated in lysogeny broth (LB) medium or modified M9 medium with d-xylose as carbon source (Supplementary Table S1). l-Lysine was added in various concentrations for biotransformation experiments. Growth experiments with knockout strains were performed in M9 medium or modified M9 medium containing l-lysine, d-lysine, or d-xylose as the only carbon source. When needed, antibiotics were added to the medium in the specified working concentrations: streptomycin (100 µg mL^−1^, Sm^100^), kanamycin (50 µg mL^−1^, Km^50^), and gentamicin (25 µg mL^−1^, Gm^25^). All media and solutions used in this study are listed in Supplementary Table S1.

### Bacterial strains and plasmids

The bacterial strains and plasmids used in this study are presented in Supplementary Table S2 and Supplementary Table S3. *Escherichia coli* DH5α strains were used for plasmid construction and plasmid propagation. As the megaplasmid pSTY of wild-type *Pseudomonas taiwanensis* VLB120 easily gets lost during genetic manipulations [40], we employed *Pseudomonas taiwanensis* VLB120∆C in our study. The strain harbors a streptomycin resistance on the megaplasmid, which ensured retention of the megaplasmid throughout our experiments [28]. Single knockout strains were used for analysis of the growth behaviour on l-lysine as the sole carbon source. *P. taiwanensis* VLB120∆C∆PVLB23330∆PVLB08625∆PVLB11490, further referred to as *P. taiwanensis* VLB120∆C∆3, was used as a chassis organism harbouring pCom10 plasmids with different KDO-encoding genes for the synthesis of hydroxy-l-lysine.

### Bacterial cultivation

Cells from cryogenic stocks of *P. taiwanensis* strains were streaked on LB agar plates and incubated overnight at 30 °C. Subsequently, 2 mL LB precultures were inoculated with single colonies and cultivated for 8 h at 30 °C and 200 rpm (2.5 cm amplitude). Next, 25 mL M9 precultures were inoculated with 250 µL of the LB precultures and incubated for 18–20 h at 30 °C and 200 rpm (2.5 cm amplitude) in baffled 250 mL Erlenmeyer flasks. The M9 precultures were used to inoculate M9 main cultures in various batch cultivations as described in the following sections.

#### Microbioreactor batch cultivations

Microbioreactor batch cultivations were either performed in the BioLector I (m2p-labs, Baesweiler, Germany) or using the System Duetz (EnzyScreen BV, Heemstede, Netherlands). Cells were cultivated as described above, harvested by centrifugation (3,220 × *g*, 4 °C, 15 min), and resuspended in fresh M9 medium to an OD_450_ of 0.2. Using the BioLector I, biotransformation experiments were performed in 48-well microplates (FlowerPlate, MTP-48-B) on a 1 mL scale, at 30 °C, 1200 rpm, and 85% humidity for 72 h. Heterologous gene expression was induced after 5 h by the addition of 1 mM isopropyl-β-d-1-thiogalactopyranoside (IPTG). At the end of cultivation, the OD_450_ was determined. The cells were separated by centrifugation (21,000 × *g*, 4 °C, 10 min), and the supernatant was stored at −20 °C until further analysis. Using the System Duetz, biotransformation experiments were performed in square 24-deepwell microplates on a 3.5 mL scale at 30 °C and 200 rpm (5 cm amplitude). Heterologous gene expression was induced by the addition of 1 mM IPTG at inoculation. Samples were taken at regular intervals. Cells were separated by centrifugation (21,000 × *g*, 4 °C, 10 min), and the supernatant was stored at −20 °C until further analysis.

#### Batch cultivation in Erlenmeyer flasks

All cultivations in Erlenmeyer flasks were performed at 30 °C and 200 rpm (2.5 cm amplitude) in a Multitron standard shaker (Infors HT, Bottmingen, Switzerland). For growth evaluations of the single knockout strains, the respective strains were cultivated overnight on LB agar plates and subsequently in 10 mL LB medium in 100 mL Erlenmeyer flasks. As knockout strains might have lost their ability to grow on l-lysine, the LB cultures were used to inoculate 10 mL M9 cultures with l-lysine as the sole carbon source, omitting the M9 precultivation step. 25 mL cultures were inoculated in 250 mL Erlenmeyer flasks at an OD_450_ of 0.2 and cultivated in M9 medium with 5 g L^−1^ l-lysine as the sole carbon source. Samples were taken at regular time intervals for the determination of bacterial growth by optical density measurement at 450 nm. For growth evaluations of the triple knockout strain *P. taiwanensis* VLB120∆C∆3 on l-lysine, d-lysine, and d-xylose, cells were cultivated overnight on LB agar plates and subsequently in 10 mL LB medium in 100 mL Erlenmeyer flasks. The LB cultures were used to inoculate 10 mL of M9 medium with l-lysine (5 g L^−1^) or d-lysine (5 g L^−1^) or 25 mL of modified M9 medium with d-xylose (20 g L^−1^) as the sole carbon source at an OD_450_ of 0.2. Growth profiles were determined with automated growth monitoring by backscatter measurement at 521 nm utilizing a cell growth quantifier (CGQ) system (Aquila Biolabs, Baesweiler, Germany).

#### Batch cultivation in stirred-tank bioreactors

For the cultivation in stirred-tank bioreactors (STRs), the DASbox system (Eppendorf SE, Hamburg, Germany) was used. Precultures were prepared as described above. Bioreactor cultures were inoculated at an OD_450_ of 0.6, and heterologous gene expression was induced immediately by the addition of 1 mM IPTG. Antifoam 204 was added in a concentration of 0.01% (v/v). Bioreactor cultivations were performed in 200 mL modified M9 medium at 30 °C, 1,000 rpm (Rushton-type impeller, 30 mm diameter) and an air volume flow rate of 3 L h^−1^ (0.25 vvm). Samples were taken at regular time intervals. Bacterial biomass was separated by centrifugation (21,000 × *g*, 4 °C, 10 min), and the supernatant was stored at −20 °C until further analysis.

### Molecular biology methods

Plasmids were isolated from *E. coli* utilizing the NucleoSpin Plasmid (no lid) kit from Macherey-Nagel GmbH & Co. KG (Düren, Germany). Purification of PCR products and enzymatic restriction reactions was performed with the NucleoSpin Gel and PCR Clean-up kit from Macherey-Nagel. PCR reactions for cloning purposes were performed with Q5 High-Fidelity DNA Polymerase 2x Master Mix (New England Biolabs Inc., Ipswich, Massachusetts, USA). The corresponding annealing temperatures were calculated with the NEB T_m_ calculator. Restriction enzymes were also purchased from New England Biolabs. Correct plasmid assembly was verified by colony PCRs, which were performed with Q5 High-Fidelity DNA Polymerase 2x Master Mix or the Taq DNA polymerase 1.1x master mix RED (Ampliqon A/S, Odense M, Denmark) after cell lysis of single colonies in alkaline polyethylene glycol (30 µL) as described elsewhere [41]. All primer sequences used in this study are presented in Supplementary Table S4. Preparation and transformation of chemically competent *E. coli* DH5α was performed according to the Inoue method [42]. Electrocompetent cells of *P. taiwanensis* VLB120 and *E. coli* DH5α λpir were prepared as described elsewhere [43, 44]. Electroporation was performed with 2 mm gap electroporation cuvettes in an EasyjecT Prima electroporator (Equibio Ltd., Kent, UK) at 2,500 V.

#### Generation of gene deletions in *P. taiwanensis* VLB120∆C strains

Putative enzymes involved in l-lysine catabolism in *P. taiwanensis* VLB120 were identified using protein BLAST searches on the NCBI website (https://blast.ncbi.nlm.nih.gov). Reference genes with known or annotated functions from *P. putida* KT2440 and *P. aeruginosa* PAO1 were obtained from the literature, and their corresponding protein sequences were retrieved from the *Pseudomonas* database (www.pseudomonas.com). Gene knockouts in l-lysine catabolism were performed by homologous recombination according to the protocol by Martínez-García and de Lorenzo [45], as described before for *P. taiwanensis* VLB120 [27, 31]. The plasmids pEMG_PVLB23330, pEMG_PVLB08625, and pEMG_PVLB11490 were constructed by restriction cloning. For that, the flanking regions (~ 500 bp) of the target knockout sequence were fused by overlap extension PCR and cloned into the pEMG backbone via the EcoRI and XbaI restriction sites. The used primers are listed in Supplementary Table S4. *E. coli* DH5α λpir was transformed with the ligated plasmid, plated on Km^50^ LB agar plates, and screened by colony PCR with the primers SPPN027/SPPN028 for correct assembly of the plasmid. Moreover, the correct nucleotide sequence was validated by Sanger sequencing. *P. taiwanensis* VLB120∆C was transformed with the pEMG plasmid and plated on LB agar plates containing Sm^100^ and Km^50^. The successful genomic integration was confirmed by resistance to kanamycin and colony PCR with respective primer pairs, based on the difference in PCR product size (PVLB_23330 deletion: PPN016/PPN019; PVLB_08625 deletion: PPN049/PPN052; PVLB_11490 deletion: PPN053/PPN056). To initiate the second recombination event via I-SceI cleavage, a positive clone was transformed with the plasmid pSW-2, and the cells were plated on LB agar plates containing Sm^100^ and Gm^25^. To identify kanamycin-sensitive clones, replica plating was performed on LB agar plates containing Sm^100^/Km^50^ and Sm^100^/Gm^25^. The successful recombination was confirmed by colony PCR based on the difference in PCR product size using the aforementioned primer pairs. For curing of the pSW-2 plasmid, cells were cultivated in LB medium without gentamicin. The successful loss of the plasmid was confirmed by replica plating on LB agar plates containing Sm^100^ and Sm^100^/Gm^25^. For the introduction of multiple knockouts, the procedure was repeated accordingly.

#### Plasmid construction for expression of KDO genes

Plasmids for expression of different KDO-encoding genes were generated by Gibson cloning [46]. Genes coding for *Caci*KDO (PPN005/PPN002), *Cpin*KDO (PPN006/PPN004), *Fjoh*KDO (PPN007/PPN008), *Nkor*KDO (PPN196/PPN197) and *Fspe*KDO (PPN198/PPN199) were amplified with the indicated primer pairs from pET-22 vectors (Supplementary Table S4). Genes coding for *Pbra*KDO, *Plum*KDO, *Bpse*KDO, and *Bpla*KDO were purchased as linear DNA fragments with overhangs for Gibson cloning from Thermo Fisher Scientific (Waltham, MA, USA). Genes coding for *Krad*KDO and *Krhi*KDO were amplified from genomic DNA of *Kineococcus radiotolerans* (DSM No. 14245) and *Kineococcus rhizosphaerae* (DSM No. 19711) with the primer pairs PPN063/PPN064 and PPN065/PPN066, respectively. The strains were retrieved from the Leibniz Institute DSMZ – German Collection of Microorganisms and Cell Cultures. The gene coding for *Lrub*KDO (Supplementary Table S5) was purchased as a linear DNA fragment from Thermo Fisher Scientific (Waltham, MA, USA) and amplified with the primers PPN200/PPN201. All PCR products were purified and used in a Gibson assembly reaction with NdeI-digested plasmid pCom10lac. Chemically competent *E. coli* DH5α cells were transformed with 5 µL of the Gibson reaction mixture and incubated for 45 min at 37 °C in 1 mL SOC medium. After that, cells were plated on LB agar plates (Km^50^) and incubated overnight at 37 °C. Correct assembly was evaluated by colony PCR with the primers SPPN01/SPPN02 and after plasmid isolation by Sanger sequencing.

### Parameter estimations for stirred-tank bioreactor experiments

The kinetic parameters of the growing-cell biotransformations in the stirred-tank bioreactors were calculated for the two growth phases, i.e., during growth on d-xylose and growth on d-xylonolactone/d-xylonate. From two independent biological replicates, the extracellular rates were estimated from measured concentration time-courses, i.e., d-xylose, l-lysine, hydroxy-l-lysine, a combined fraction of d-xylonolactone and d-xylonate, and biomass (Supplementary Tables S6 and S7). The concentrations used for the determination of extracellular rates were estimated by fitting the concentration to a mathematical model assuming exponential growth and constant yields during cultivation. Regression and parameter estimations were performed using gPROMS^®^ Process (Academic) 2.1.1 from Siemens (Munich, Germany). A detailed description of the model and parameter estimations is presented in the Supplementary Information (Sect.  Model Description).

### Analytical methods

#### Biomass quantification

Microbial biomass concentrations were determined using a correlation of cell dry weight (CDW) and the optical density of a cell suspension at 450 nm (OD_450_). OD measurements were performed with a Libra S11 spectrophotometer (Biochrom Ltd., Cambridge, UK). When necessary, samples were diluted in PBS prior to the measurement. An OD_450_ of 1 corresponded to 0.2049 g_CDW_ L^−1^ for *P. taiwanensis* VLB120∆C grown on d-xylose [31].

#### Quantification of d-xylose and d-xylonolactone/d-xylonate

Concentrations of d-xylose and d-xylonolactone/d-xylonate were determined by HPLC, utilizing an Agilent 1260 Infinity HPLC system (Agilent, Santa Clara, CA, USA) with a Metab-AAC column (300 × 7.8 mm, 10 μm particle size, ISERA GmbH, Düren, Germany) in combination with a Metab-AAC guard column (10 × 7.8 mm, 10 μm particle size). HPLC analysis was run in isocratic mode using 5 mM H_2_SO_4_ as mobile phase at a flow rate of 0.8 mL min^−1^ and a column oven temperature of 40 °C for 28 min. Peaks were detected using a variable wavelength detector (G1314B 1260 VWD VL, Agilent, Santa Clara, CA, USA) at a wavelength of 210 nm and a refractive index (RI) detector (G1362A 1260 RID, Agilent, Santa Clara, CA, USA). Using the applied HPLC conditions, d-xylose and d-xylonolactone/d-xylonate elute at the same time. However, d-xylose was only detected with the RI detector, and d-xylonolactone/d-xylonate were detected as one peak with the RI and the UV detector. The concentration of d-xylonolactone/d-xylonate was determined from the UV detector signal. The contribution of d-xylonolactone/d-xylonate to the combined RI signal was calculated based on its UV-determined concentration, allowing the concentration of d-xylose to be obtained by subtracting the corresponding RI response of d-xylonolactone/d-xylonate from the total RI peak area.

#### Quantification of l-lysine and hydroxy-l-lysine

Detection and quantification of l-lysine and hydroxy-l-lysine was performed on a Shimadzu HPLC system consisting of a SCL-40 system controller, a DGU-403 degassing unit, two LC-20AT pumps, a SIL-40 autosampler, and a CTO-40 C column oven (Shimadzu Germany GmbH, Duisburg, Germany) coupled to a Corona charged aerosol detector (ESA Biosciences, Inc., USA). Chromatographic separation was performed using a zwitterionic hydrophilic interaction liquid chromatography (HILIC) column SeQuant ZIC-pHILIC (150 × 4.6 mm, 5 μm particle size, 200 Å pore size; Merck KGaA, Darmstadt, Germany) in combination with a SeQuant ZIC-pHILIC guard column (20 × 2.1 mm, 5 μm particle size, 200 Å pore size). HPLC analysis was performed in isocratic mode using a 70% (v/v) acetonitrile and 30% (v/v) ammonium acetate solution (50 mM, pH 4.0) with a flow rate of 0.8 mL min^−1^ for 28 min and a column oven temperature of 40 °C. Quantification was performed by external calibration with standard samples of l-lysine (Sigma-Aldrich, St. Louis, MI, USA) and 5-hydroxy-dl-lysine hydrochloride (Carbosynth, Compton, UK).

## Results

### Inactivation of endogenous l-lysine catabolism

**Fig. 2:**
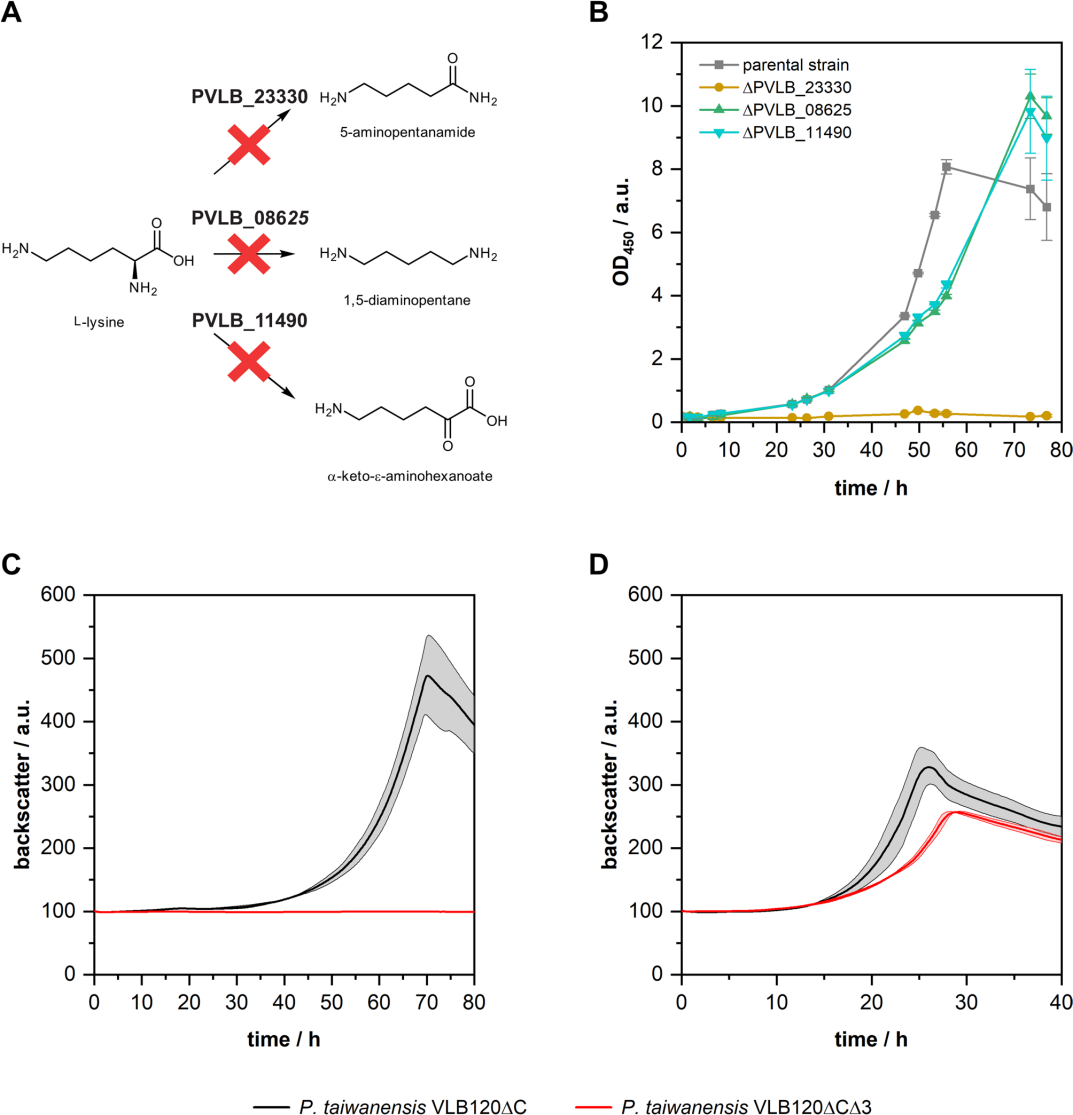
Knockout of l-lysine catabolism of *P. taiwanensis* VLB120. The genes PVLB_23330, PVLB_08625, and PVLB_11490, each putatively encoding the initial reaction of a l-lysine catabolic pathway in *P. taiwanensis* VLB120, were identified as homologs of *davB*, *ldcC*, and *aruH* from *P. putida* KT2440 (**A**). Growth of the single gene knockout strains and the parental strain *P. taiwanensis* VLB120ΔC on l-lysine as sole carbon source (**B**). Strains were cultivated in M9 medium (5 g L^−1^ l-lysine, 30 °C, 200 rpm). Growth of *P. taiwanensis* VLB120ΔC and the triple knockout strain *P. taiwanensis* VLB120ΔCΔ3 on l-lysine (**C**) and d-lysine (**D**). Cultivations were performed in 10 mL M9 medium supplemented with 5 g L^−1^ of either l-lysine or d-lysine as the sole carbon source (30 °C, 200 rpm). Growth was monitored using the Cell Growth Quantifier (CGQ) system (Aquila Biolabs). Mean values and error bars (standard deviations) are calculated from two independent biological replicates

Pseudomonads are known to possess multiple pathways for the degradation of l-lysine [32]. Hence, to enable hydroxylation of l-lysine in *P. taiwanensis* VLB120, the native catabolic pathways need to be eliminated to avoid loss of the substrate l-lysine, and potentially also the product hydroxy-l-lysine. Therefore, we analyzed the genome of *P. taiwanensis* VLB120 to identify l-lysine catabolic routes and potential targets for gene knockouts. We searched for genes of all four known catabolic routes by reference to the genes from *Pseudomonas putida* KT2440 and *Pseudomonas aeruginosa* PAO1 and reconstructed the pathways in silico (Supplementary Figure S1). We found three potential pathways, with the initial reactions encoded by homologs of the lysine 2-monooxygenase-encoding gene *davB* (PVLB_23330), the lysine decarboxylase-encoding gene *ldcC* (PVLB_08625), and the aminotransferase-encoding gene *aruH* (PVLB_11490) (Fig. 2A). While we could not find a genomic ortholog of *alr* coding for the lysine racemase, we identified an ortholog of *dadX* (PVLB_24950), coding for another amino acid racemase. However, l-lysine is not a substrate of DadX from *P. putida* KT2440 [35]. Thus, to prevent degradation of l-lysine during biotransformations, we cut off the three existing catabolic pathways by knocking out the genes PVLB_23330, PVLB_08625, and PVLB_11490. We investigated the growth of *P. taiwanensis* VLB120∆C and the deletion mutants in M9 minimal medium with l-lysine as the sole carbon source (Fig. 2B). *P. taiwanensis* VLB120∆C was able to grow on l-lysine, whereas the knockouts of PVLB_08625 and PVLB_11490 led to a reduced growth rate of the respective mutants. The deletion of PVLB_23330 resulted in a complete loss of growth. This demonstrated that PVLB_23330 is involved in growth on l-lysine. Although this was not clearly evident for PVLB_08625 and PVLB_11490, we decided to delete all three genes in a single strain as a precaution to enable maximum substrate utilization for the desired hydroxylation reaction. Therefore, we created the triple knockout strain *P. taiwanensis* VLB120∆C∆PVLB23330∆PVLB08625∆PVLB11490, further referred to as *P. taiwanensis*∆C∆3. Consistent with the phenotype observed for the ∆PVLB23330 single knockout strain, the triple knockout strain was unable to grow on l-lysine as sole carbon source (Fig. 2C). In contrast, growth experiments on d-lysine revealed that both the parental strain and the triple knockout strain can grow on d-lysine as sole carbon source (Fig. 2D). These results indicate the presence of an active catabolic pathway for d-lysine, even though the absence of lysine racemase prevents the interconversion of l-lysine to d-lysine. Lastly, we tested the growth performance of the engineered strain on d-xylose in comparison to the parental strain to ensure that *P. taiwanensis*∆C∆3 was still able to grow on d-xylose in an uncompromised manner (Supplementary Figure S2). As no difference in growth behavior was detectable, the genetic modifications appeared not to have changed the strain’s ability for growth on d-xylose.

### Screening of *P. taiwanensis* VLB120∆C∆3 whole-cell biocatalysts

**Fig. 3:**
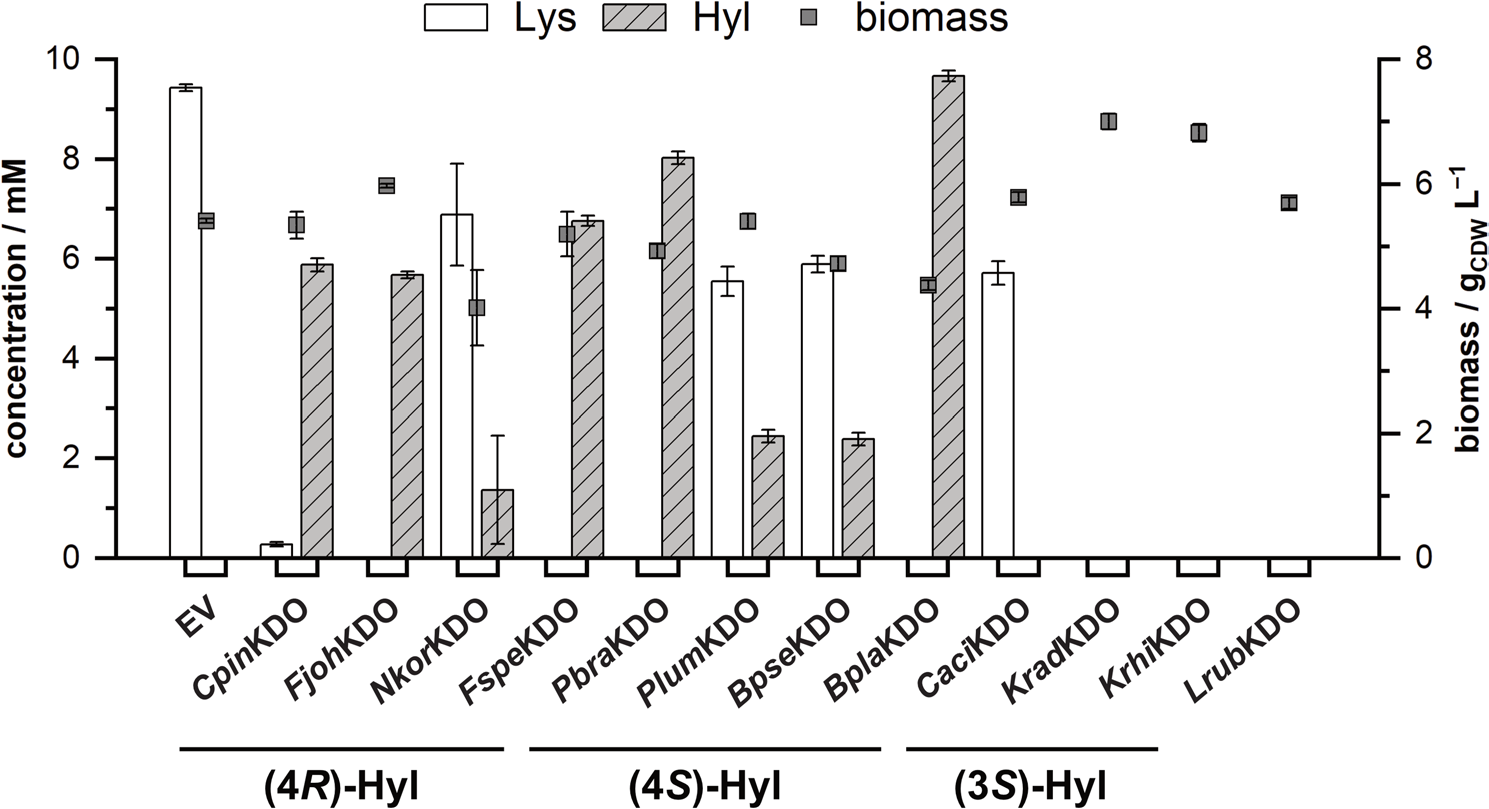
Screening of whole-cell biocatalysts for hydroxylation of l-lysine. Strains expressing KDO genes are arranged according to their respective product (4*R*)-4-hydroxy-l-lysine, (4*S*)-4-hydroxy-l-lysine, or (3*S*)-3-hydroxy-l-lysine. Depicted are the concentrations of l-lysine (Lys), hydroxy-l-lysine (Hyl), and bacterial biomass after 72 h of cultivation. Cultivations of *P. taiwanensis* VLB120∆C∆3 harboring plasmids coding for different KDOs or the empty-vector control (EV) were performed in modified M9 medium (20 g L^−1^ d-xylose, 10 mM l-lysine) at 30 °C on a 1 mL scale in a BioLector. Gene sequences coding for the KDOs originate from *Chitinophaga pinensis* (*Cpin*KDO), *Flavobacterium johnsoniae* (*Fjoh*KDO), *Niastella koreensis* (*Nkor*KDO), *Flavobacterium* species (*Fspe*KDO), *Polyangium brachysporum* (*Pbra*KDO), *Photorhabdus luminescens* (*Plum*KDO), *Burkholderia pseudomallei* (*Bpse*KDO), *Burkholderia plantarii* (*Bpla*KDO), *Catenulispora acidiphila* (*Caci*KDO), *Kineococcus radiotolerans* (*Krad*KDO), *Kineococcus rhizosphaerae* (*Krhi*KDO), and *Leifsonia rubra* (*Lrub*KDO). Mean values and error bars (standard deviation) are calculated from three independent biological replicates.

In a previous study, we investigated various KDOs in cell-free and *E. coli*-based whole-cell biotransformations [47]. To test *P. taiwanensis* VLB120∆C∆3 for the synthesis of hydroxy-l-lysine, our genetically engineered strain was transformed with plasmids harboring genes coding for different KDOs, resulting in twelve whole-cell biocatalysts. The whole-cell biocatalysts can be divided into three groups in dependence on the formed product, i.e., (4*R*)-4-hydroxy-l-lysine, (4*S*)-4-hydroxy-l-lysine, or (3*S*)-3-hydroxy-l-lysine. *Cpin*KDO, *Fjoh*KDO, *Nkor*KDO, and *Fspe*KDO are known to produce (4*R*)-4-hydroxy-l-lysine [10]. In the original study by Baud et al., these enzymes were referred to as KDO2, KDO3, KDO4, and KDO5, respectively [10]. For clarity and consistency, we named the enzymes as in our previous study, using abbreviations of the bacterial strains from which the respective enzyme originate [47]. *Caci*KDO (also referred to as KDO1 [10]) and *Krad*KDO (also referred to as K3H-1 [48] or K3H [49]) have been shown to produce (3*S*)-3-hydroxy-l-lysine, and *Krhi*KDO is hypothesized to catalyze the formation of the same product [47], produce (3*S*)-3-hydroxy-l-lysine. *Pbra*KDO (also referred to as GlbB [4]), produces (4*S*)-4-hydroxy-l-lysine, and its homologs *Plum*KDO, *Bpse*KDO, and *Bpla*KDO are hypothesized to yield the same product [47]. The KDO from *Leifsonia rubra* (*Lrub*KDO) shares 54.1% sequence identity with *Krad*KDO and 42.6% with *Caci*KDO, and has not been investigated before. The constructed strains were cultivated in M9 medium with d-xylose as substrate for growth and supply of the cosubstrate α-KG, and l-lysine as substrate for hydroxylation. The concentrations of l-lysine and hydroxy-l-lysine and the generated biomass were determined after 72 h of cultivation (Fig. 3).

In our experiments, eight out of twelve strains produced hydroxy-l-lysine in quantifiable concentrations. The empty-vector control showed only a small decrease (~ 6%) of l-lysine in the supernatant. All strains for the synthesis of (4*R*)-4-hydroxy-l-lysine showed product formation. The biotransformations with the strains harboring KDOs from *Chitinophaga pinsensis*, *Flavobacterium johnsoniae*, and *Flavobacterium* species resulted in almost complete conversion of l-lysine and hydroxy-l-lysine concentrations between 5.7 and 6.8 mM. The strain with the KDO from *Niastella koreensis* had ~ 7 mM of unused l-lysine left at the end of cultivation. Thus, product formation was less than with the other three strains. All strains for the synthesis of (4*S*)-4-hydroxy-l-lysine showed product formation as well. However, for *P. taiwanensis* VLB120∆C∆3 pCom10lac_*Plum*KDO and *P. taiwanensis* VLB120∆C∆3 pCom10lac_*Bpse*KDO, there were still ~ 6 mM l-lysine left at the end of cultivation. l-Lysine was entirely depleted in the cultivations with the strains harboring pCom10lac_*Pbra*KDO and pCom10lac_*Bpla*KDO, resulting in the highest concentrations of 8.0 mM and 9.7 mM hydroxy-l-lysine, respectively. The whole-cell biotransformations with the strains for the synthesis of (3*S*)-3-hydroxy-l-lysine resulted in no product formation. In the cultivation with *P. taiwanensis* VLB120∆C∆3 pCom10lac_*Caci*KDO, there were 5.7 mM l-lysine left in the medium, while l-lysine was completely taken up by the two other strains. The cultivations of *P. taiwanensis* VLB120∆C∆3 pCom10lac_*Krad*KDO and *P. taiwanensis* VLB120∆C∆3 pCom10lac_*Krhi*KDO showed an increased biomass concentration at the end of cultivation. Moreover, as l-lysine was only taken up to a high degree when genes coding for KDOs were expressed, the lack of product might be a result of product degradation. Thus, in contrast to (4*R*)- and (4*S*)-4-hydroxy-l-lysine, (3*S*)-3-hydroxy-l-lysine seems to be metabolized by *P. taiwanensis* VLB120. Additionally, this suggests that *Lrub*KDO also performs the hydroxylation of the C-3 position of l-lysine. To further evaluate the potential of the whole-cell biocatalysts, we selected and tested the three most promising strains and performed biotransformations using increased substrate concentrations.

###  Growing-cell biotransformations of selected strains with increased concentrations of l-lysine

**Fig. 4:**
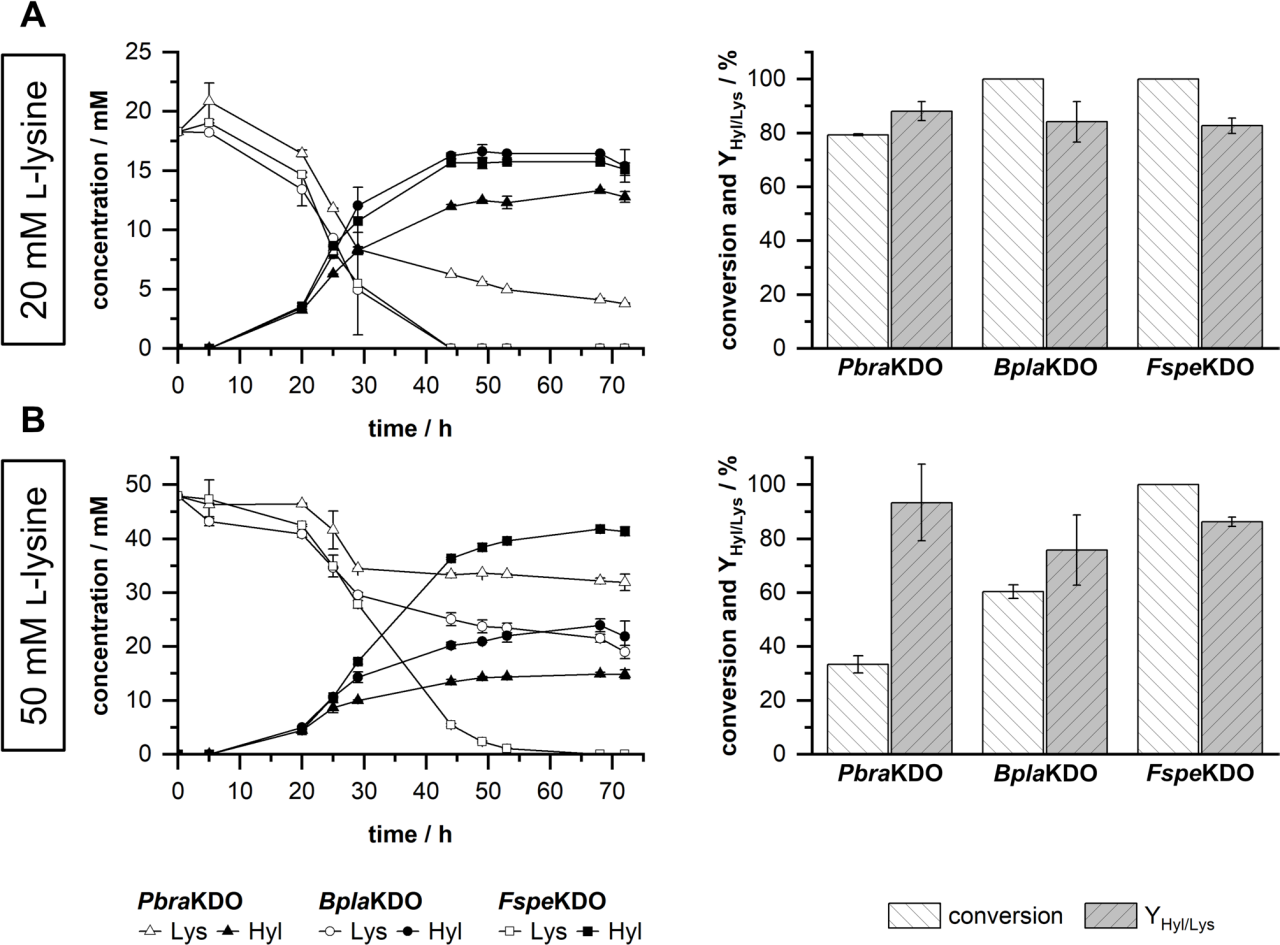
Growing-cell biotransformations of selected strains with 20 mM and 50 mM l-lysine. Biotransformations were performed with *P. taiwanensis* VLB120∆C∆3 expressing KDO genes from *Polyangium brachysporum* (*Pbra*KDO), *Burkholderia plantarii* (*Bpla*KDO), and *Flavobacterium* species (*Fspe*KDO). Depicted are the progress curves of substrate l-lysine (Lys) and product hydroxy-l-lysine (Hyl), as well as the conversion and molar yield of hydroxy-l-lysine on l-lysine (Y_Hyl/Lys_) for biotransformations with 20 mM initial l-lysine (**A**) and 50 mM initial l-lysine (**B**). Conversion and molar yield of hydroxy-l-lysine on lysine (Y_Hyl/Lys_) were calculated for values obtained at 72 h of cultivation. Growing-cell biotransformations were performed in modified M9 medium (20 g L^−1^ d-xylose) using System Duetz 24-deepwell microplates on a 3.5 mL scale, at 30 °C, and 200 rpm. Mean values and error bars (standard deviation) are calculated from two independent biological replicates

The strains with heterologous expression of KDO genes from *Polyangium brachysporum (Pbra*KDO*)*, *Burkholderia plantarii* (*Bpla*KDO), and *Flavobacterium* species (*Fspe*KDO) achieved complete conversion of l-lysine and the highest concentrations of hydroxy-l-lysine (Fig. 3). Therefore, we next performed biotransformations using these three strains with 20 mM and 50 mM l-lysine to evaluate the biocatalyst performance with increased substrate concentrations (Fig. 4). With initial concentrations of 20 mM l-lysine, strains expressing KDOs from *Flavobacterium* species and *Burkholderia plantarii* resulted in full conversion of l-lysine and molar yields of hydroxy-l-lysine on l-lysine (Y_Hyl/Lys_) of 82.6% and 84.2%, respectively (Fig. 4A). In contrast to that, only *P. taiwanensis* VLB120∆C∆3 pCom10lac_*Fspe*KDO was able to fully convert l-lysine with 50 mM initial l-lysine and resulted in a Y_Hyl/Lys_ of 86.3% (Fig. 4B). Growing-cell biotransformations with *P. taiwanensis* VLB120∆C∆3 pCom10lac_*Pbra*KDO and *P. taiwanensis* VLB120∆C∆3 pCom10lac_*Bpla*KDO resulted in conversions of 33.4% and 60.4% with 50 mM initial l-lysine and Y_Hyl/Lys_ values of 93.4% and 75.8%, respectively. In summary, *P. taiwanensis* VLB120∆C∆3 pCom10lac_*Fspe*KDO outperformed the two other strains, and we set out to further characterize and optimize the growing-cell biotransformation with this strain in the following steps.

### Influence of l-lysine and Fe^2+^ on growing-cell biotransformations with *P. taiwanensis* VLB120∆C∆3 pCom10lac_*Fspe*KDO

**Fig. 5:**
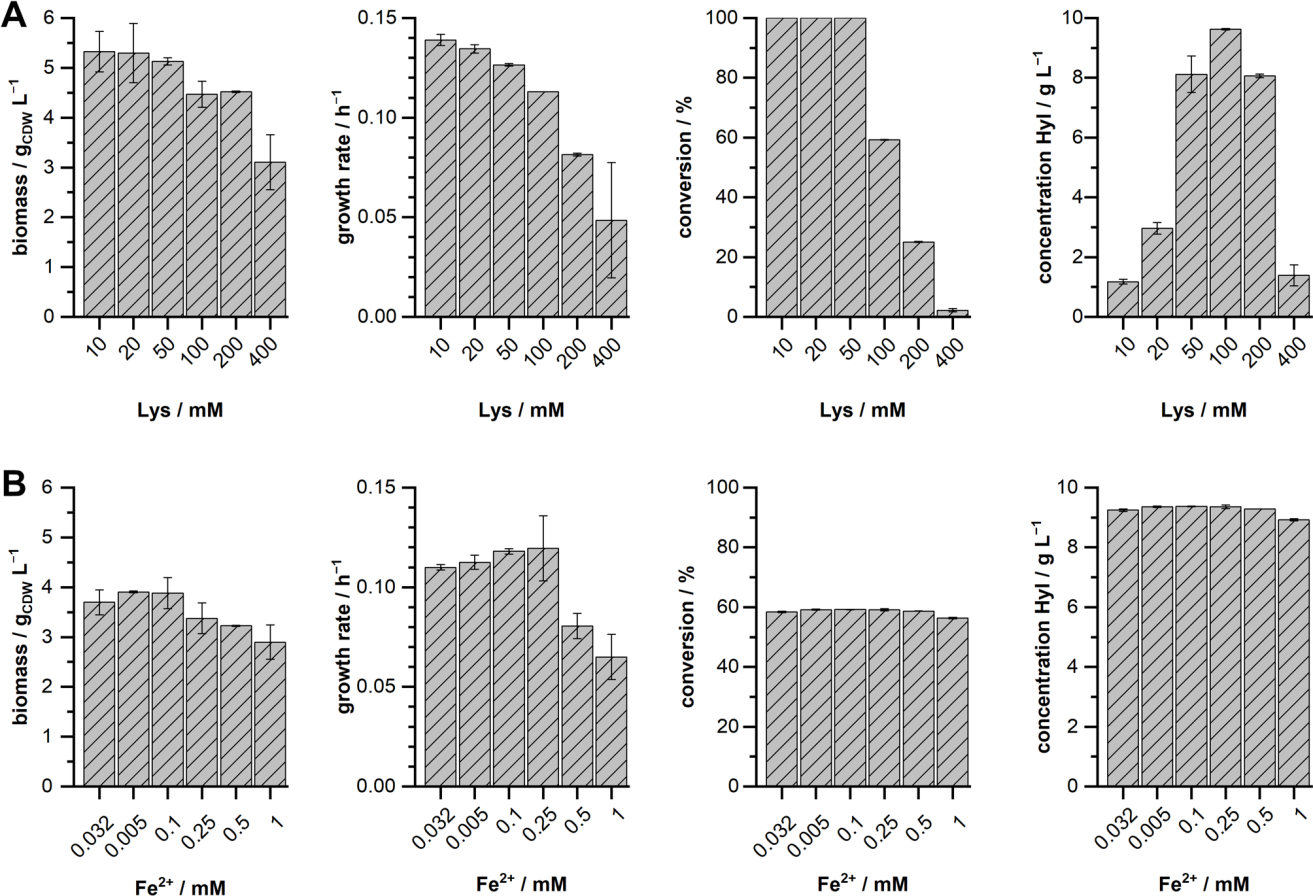
Influence of the concentrations of l-lysine and Fe^2+^ on growing-cell biotransformations with *P. taiwanensis* VLB120∆C∆3 pCom10lac_*Fspe*KDO. Key parameters of the growing-cell biotransformations, i.e., final biomass, growth rate, substrate conversion, and product concentration, were determined for experiments with varying l-lysine concentrations (**A**) and varying concentrations of the metal cofactor Fe^2+^ by supplementation of FeSO_4_ (**B**). Cultivations were performed for 72 h in modified M9 medium (20 g L^−1^ d-xylose) at 30 °C on a 1 mL scale in a BioLector. For the experiments with varying l-lysine concentrations, Fe^2+^ was supplemented at 32 µM. For the experiments with varying Fe^2+^ concentrations, l-lysine was supplemented at 100 mM. Mean values and error bars (standard deviation) are calculated from two independent biological replicates

Our previous experiments with increased substrate concentrations revealed *P. taiwanensis* VLB120∆C∆3 pCom10lac_*Fspe*KDO as the most promising whole-cell biocatalyst. Therefore, we used this strain to explore the influence of elevated substrate concentrations and the concentration of the cofactor metal ion Fe^2+^ on the biotransformation. We performed growing-cell biotransformations with varying concentrations of the substrate l-lysine (10 – 400 mM) and varying concentrations of Fe^2+^ (32 µM – 1 mM) and studied the impact on biomass generation, growth rate, conversion of l-lysine, and the final hydroxy-l-lysine concentration (Fig. 5).

The final biomass concentration was slightly reduced between 100 and 200 mM l-lysine and more substantially at 400 mM l-lysine. However, increasing substrate concentrations reduced the observed growth rates already below 400 mM l-lysine (Fig. 5A). Up to 50 mM l-lysine, *P. taiwanensis* VLB120∆C∆3 pCom10lac_*Fspe*KDO was able to completely convert the substrate until the end of the cultivation, as observed in our previous experiments (Fig. 4B). At 100 mM l-lysine, the strain was still able to convert approximately 60% of the substrate. The highest product concentration of ~ 9.6 g L^−1^ was detected with a starting concentration of 100 mM l-lysine. Higher and lower initial starting concentrations of l-lysine resulted in decreased product concentrations. The tested variation of Fe^2+^ showed only low overall effects (Fig. 5B). Increasing concentrations of Fe^2+^ led to slightly reduced final biomass concentrations. In comparison to the standard concentration of Fe^2+^ (32 µM), we observed reduced growth rates of 0.08 h^−1^ and 0.06 h^−1^ at 0.5 mM and 1 mM Fe^2+^, respectively. The variation of Fe^2+^ did not affect the conversion and the final product concentrations.

### Production of hydroxy-l-lysine in stirred-tank bioreactors

**Fig. 6:**
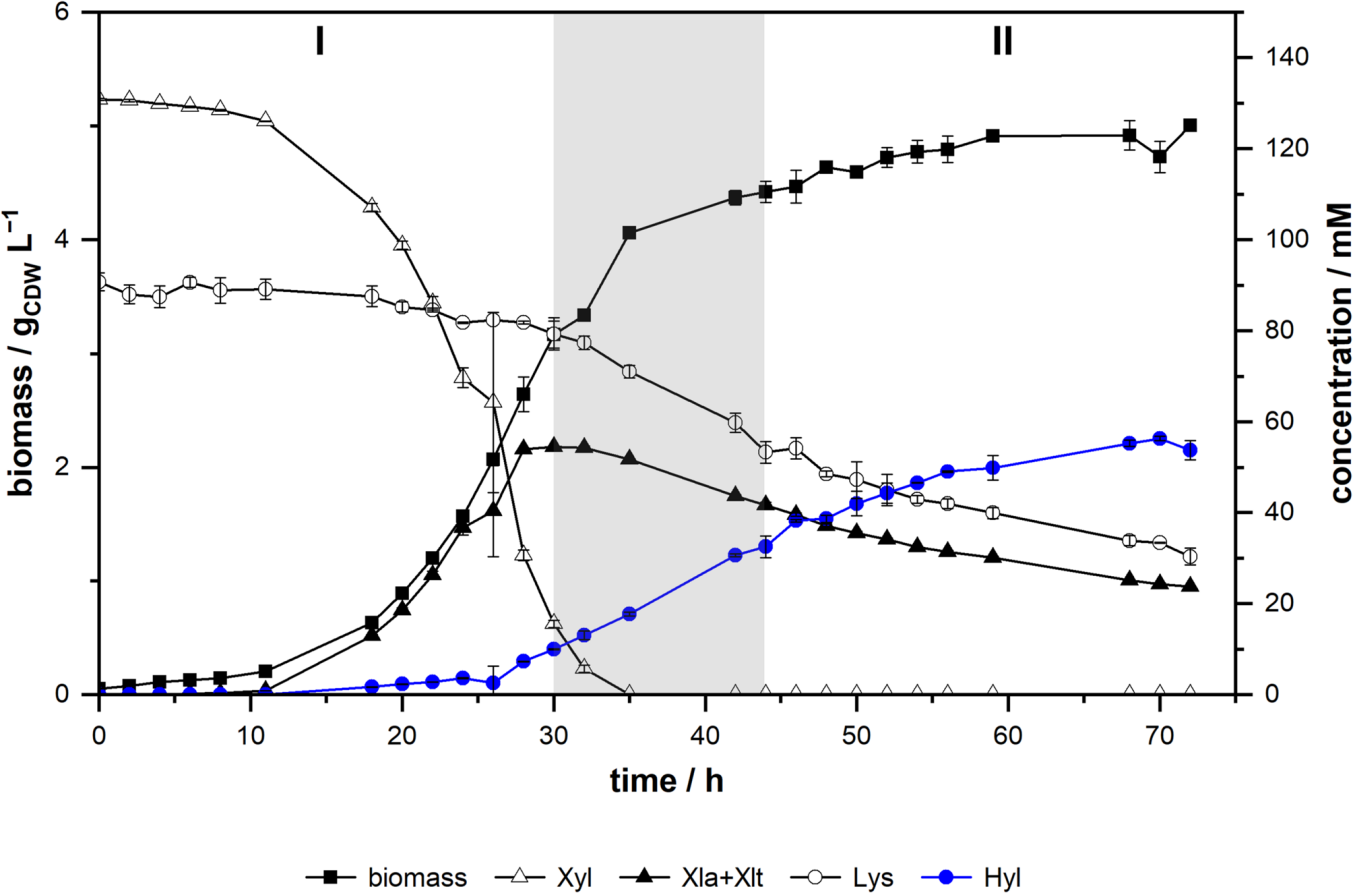
Growing-cell biotransformation with *P. taiwanensis* VLB120ΔCΔ3 pCom10lac_*Fspe*KDO in a stirred-tank bioreactor. The biotransformation was performed in 200 mL modified M9 medium (100 mM l-lysine, 20 g L^−1^ d-xylose) in a stirred-tank bioreactor over 72 h at 30 °C, 1,000 rpm, and an aeration rate of 3 L h^−1^. The two observed growth phases are labeled I and II, and the transition phase is marked in grey. Mean values and error bars (standard deviation) are calculated from two independent biological replicates. Xyl – d-xylose, Xla+Xlt – combined concentration of Weimberg pathway intermediates d-xylonolactone and d-xylonate, Lys – l-lysine, Hyl – hydroxy-l-lysine

In the next step, we transferred the process from a microbioreactor scale to a stirred-tank bioreactor to characterize the biotransformation in detail under a well-controlled technical environment and under more industrially relevant process conditions. We performed batch cultivations with *P. taiwanensis* VLB120∆C∆3 pCom10lac_*Fspe*KDO, both with and without the substrate l-lysine, to gain further insights into the process characteristics. The biomass profile during cultivation indicates two distinct growth phases (Fig. 6, Supplementary Figure S3). In the first phase, d-xylose is taken up, and exponential growth is visible with a growth rate of 0.125 h^−1^ (Table 1). Simultaneously, d-xylonolactone/d-xylonate accumulates in the medium. During this phase, the l-lysine concentration slowly decreases and hydroxy-l-lysine begins to accumulate in the medium. In the second growth phase, d-xylose is depleted, and the cells take up d-xylonolactone/d-xylonate for growth, resulting in a linear growth profile. Although the main production occurred in the second phase, due to the higher biomass concentration, l-lysine was taken up faster in the first growth phase, as indicated by the specific l-lysine uptake rate (Table 1). In accordance with that, the specific hydroxy-l-lysine production rate was higher in the first growth phase than in the second phase. However, the yield of hydroxy-l-lysine based on biomass (Y_Hyl/X_) was significantly increased in the second growth phase (36.12 ± 4.31 mmol g_CDW_^−1^) compared to the first growth phase (2.92 ± 0.22 mmol g_CDW_^−1^). In comparison to the cultivations without l-lysine, the biotransformations showed a reduced growth rate and a reduced d-xylose uptake rate (Table 1).

To assess the overall performance of the biotransformation, we calculated key performance metrics for the complete biotransformation process (Table 2). The stirred-tank biotransformations resulted in a concentration of 8.7 ± 0.3 g L^−1^ (53.8 ± 2.1 mM) hydroxy-l-lysine with a space-time yield of 98.6 ± 3.4 mg L^−1^ h^−1^. The yield of hydroxy-l-lysine on d-xylose was 0.38 ± 0.01 mol mol^−1^. However, when subtracting the amount of d-xylose that ended up as the intermediates d-xylonolactone/d-xylonate, we calculated a net yield of 0.48 ± 0.02 mol mol^−1^ hydroxy-l-lysine on d-xylose. That means, roughly every second molecule of d-xylose which was converted beyond d-xylonate and entered the central carbon metabolism was used for the biotransformation reaction.

**Table 1:** Key performance metrics of *P. taiwanensis* VLB120ΔCΔ3 pCom10lac_*Fspe*KDO in stirred-tank bioreactor cultivations

	+ l-lysine			− l-lysine	
	Phase I	Phase II		Phase I	Phase II
µ / h^−1^	0.125 ± 0.003	0.007 ± 0.001		0.155 ± 0.002	0.007 ± 0.000
r_Xyl_ / mmol g_CDW_ h^−1^	−4.88 ± 0.16	n.a.		−6.06 ± 0.24	n.a.
r_XlaXlt_ / mmol g_CDW_ h^−1^	22.89 ± 0.22	−0.17 ± 0.02		21.89 ± 0.57	−0.13 ± 0.02
Y_Hyl/X_ / mmol g_CDW_^−1^	2.92 ± 0.22	36.12 ± 4.31		n.a.	n.a.
r_Hyl_ / mmol g_CDW_ h^−1^	0.366 ± 0.028	0.244 ± 0.036		n.a.	n.a.
r_Lys_ / mmol g_CDW_ h^−1^	−0.43 ± 0.05	−0.21 ± 0.05		n.a.	n.a.

Cultivations / growing-cell biotransformations were performed in 200 mL M9 medium (20 g L^−1^ d-xylose) with or without 100 mM l-lysine in a stirred-tank bioreactor (30 °C, 1,000 rpm, and an aeration rate of 3 L h^−1^). Growth phases are divided as depicted in Fig. 6. The extracellular rates µ, r_Xyl_, r_XlaXlt_, and r_Hyl_ were estimated separately for the two phases by fitting the concentration profiles to an exponential growth model, assuming constant yields in the respective growth phases. The standard error of the extracellular rates was determined using the appropriate error propagation. µ – growth rate, r_Xyl_ – specific d-xylose uptake rate, r_XlaXlt_ – specific combined d-xylonolactone and d-xylonate production (uptake, Phase II) rate, r_Hyl_ – specific hydroxy-l-lysine production rate, r_Lys_ – specific l-Lysine uptake rate, Y_Hyl/X_ – yield of hydroxy-l-lysine on biomass, n.a. – not applicable

**Table 2:** Overall performance metrics of the growing-cell biotransformations with *P. taiwanensis* VLB120ΔCΔ3 pCom10lac_*Fspe*KDO performed in stirred-tank bioreactors

Parameter	Value
c_Hyl_	8.7 ± 0.3 g L^−1^ (53.8 ± 2.1 mM)
Y_Hyl/X_	1.68 ± 0.07 g g_CDW_^−1^
Y_Hyl/Lys_	0.89 ± 0.10 mol mol^−1^
Y_Hyl/Xyl_	0.38 ± 0.01 mol mol^−1^
Y_Hyl/Xyl,net_	0.48 ± 0.02 mol mol^−1^
STY	98.6 ± 3.4 mg L^−1^ h^−1^

*c*_Hyl_ – concentration of hydroxy-l-lysine, *Y*_Hyl/X_ – yield of hydroxy-l-lysine on biomass, *Y*_Hyl/Lys_ – yield of hydroxy-l-lysine on l-lysine, *Y*_Hyl/Xyl_ – yield of hydroxy-l-lysine on d-xylose, *Y*_Hyl/Xyl,net_ – yield of hydroxy-l-lysine on d-xylose corrected for lost d-xylose as pathway intermediates d-xylonolactone/d-xylonate, STY – space-time yield. Metrics were corrected for changes in the reaction volume due to sample withdrawal. Mean values and standard deviations are calculated from two independent biological replicates

## Discussion

*P. taiwanensis* VLB120 constitutes an attractive biochemical chassis for the utilization of the renewable carbon source d-xylose in sustainable bioprocesses. In the present study, a novel bioprocess for the hydroxylation of l-lysine with *P. taiwanensis* VLB120 as a whole-cell biocatalyst was engineered, whereby d-xylose serves as the sole carbon and energy source. In order to establish this novel *P. taiwanensis* VLB120 chassis, we first investigated the endogenous l-lysine catabolism for potential gene targets to avoid the consumption of l-lysine for biomass formation. Our analyses revealed that *P. taiwanensis* VLB120 has genes for three potential routes for the degradation of l-lysine. A route via lysine decarboxylase (PVLB_08625), a second route via lysine 2-monooxygenase (PVLB_23330), and a third route via an aminotransferase (PVLB_11490). Our results suggest that the route via lysine 2-monooxygenase is the primary catabolic pathway for *P. taiwanensis* VLB120, as the knockout of PVLB_23330 led to a complete loss of growth on l-lysine, while knockouts of PVLB_08625 and PVLB_11490 only resulted in altered growth behavior. As the growth behavior of the two strains carrying deletions of PVLB_08625 and PVLB_11490 may also result from secondary effects unrelated to l-lysine catabolism, the actual utilization of the two metabolic routes still requires conclusive experimental confirmation. Nevertheless, *Pseudomonas* strains are known to harbor multiple parallel pathways for l-lysine catabolism, with one or several of these pathways being dominant under specific conditions [32]. Interestingly, while the triple knockout strain *P. taiwanensis* VLB120ΔCΔ3 was unable to grow on l-lysine as sole carbon source, it was able to grow on d-lysine (Fig. 2C-D). This observation is in accordance with our genomic analysis and supports the hypothesis that the catabolic route starting from d-lysine is present, but due to the missing racemase, l-lysine cannot be converted to d-lysine. In *P. putida* KT2440, the conversion of l-lysine to d-lysine is performed by the broad-spectrum amino acid racemase Alr [35]. For this strain, it was shown that *alr* is not essential, but that deletion of the gene leads to growth defects on l-lysine and l-arginine [50].

While catabolism of l-lysine is well studied in *P. putida* and *P. aeruginosa*, there is only scarce information about catabolic routes for hydroxy-l-lysine. Experiments in *P. fluorescens* showed that 5-hydroxy-l-lysine is catabolized via the monooxygenase pathway and the racemase pathway from l-lysine catabolism [37]. This suggests that the l-lysine catabolic pathways might also convert other isomers of hydroxy-l-lysine. The triple knockout strain *P. taiwanensis* VLB120ΔCΔ3 enabled hydroxylation of l-lysine, although the converted l-lysine was not always recovered as a product. To some extent, this can be explained by the fact that l-lysine is used in other pathways, such as protein synthesis. However, we cannot exclude that *P. taiwanensis* VLB120ΔCΔ3 has other enzymes present that degrade l-lysine or hydroxy-l-lysine. This becomes even more apparent as we were only able to obtain 4-hydroxy-l-lysine in the biotransformations but not 3-hydroxy-l-lysine. Hence, it is likely that there is at least another enzyme that is able to use 3-hydroxy-l-lysine as substrate but not 4-hydroxy-l-lysine. Potential candidates might be enzymes that also have side activity for l-lysine, such as ornithine decarboxylase [51, 52]. Identification of the responsible enzyme(s) and knockout of the respective gene(s) might facilitate even synthesis of 3-hydroxy-l-lysine in the future.

In addition to the potential degradation of l-lysine and hydroxy-l-lysine, the observed differences in the performance of the whole-cell biocatalysts during the screening may be attributed to variations in the enzymatic properties of the KDOs (e.g., K_m_, k_cat_). Furthermore, differences in gene expression levels may have contributed to the observed differences in whole-cell biocatalyst performance. In our experiments, strains expressing KDO genes showed greater apparent uptake of l-lysine than the empty-vector control strain. One possible reason might be the increased intracellular consumption of l-lysine, creating a metabolic sink that drives the import. By contrast, the empty-vector control exhibited only limited uptake, resulting in higher residual l-lysine concentrations in the medium. The specific substrate consumption of l-lysine was roughly ten times slower compared to the specific uptake rate of d-xylose in the first growth phase. Only in the second growth phase, the uptake of d-xylonate/d-xylonolactone and l-lysine correlated approximately stoichiometrically with the generation of hydroxy-l-lysine. As the KDO reaction competes with other cellular pathways for the α-KG pool, fine-tuning the d-xylose and l-lysine substrate consumption might lead to increased d-xylose utilization for the biotransformation. Improving the substrate transport has already been shown to enhance the performance of Fe^2+^/α-ketoglutarate-dependent oxygenase-based whole-cell biocatalysts. For example, overexpression of the proline transporter gene *putP* led to an increased specific hydroxylation rate of l-proline with proline hydroxylase in *E. coli* [16]. Moreover, in *E. coli*-based whole-cell biocatalysts utilizing the KDO from *Kineococcus radiotolerans*, overexpression of *argT* and *cadB* from *E. coli* resulted in significantly increased titers of (3*S*)-3-hydroxy-l-lysine [49]. ArgT is a periplasmic binding protein, belonging to the ABC transporter complex HisPMQ-ArgT, responsible for lysine/arginine/ornithine transport, and CadB is a cadaverine/lysine antiporter [49]. *P. taiwanensis* VLB120 harbors orthologs of the two ABC transporter systems which have been reported for l-lysine import in *P. putida* KT2440 [39]. The one being composed of PVLB_23890 (PP_0280, 96.51% identity), PVLB_23885 (PP_0281, 95.65% identity), PVLB_23880 (PP_0282, 96.02% identity) and PVLB_23875 (PP_0283, 97.67% identity) and the other one being composed of PVLB_17555 (PP_4483, 98.03% identity), PVLB_17560 (PP_4484, 95.26% identity), PVLB_17565 (PP_4485, 96.07% identity) and PVLB_17570 (PP_4486, 95.40% identity). However, the contribution of these transporters to the transport of l-lysine in *P. taiwanensis* VLB120 has not yet been experimentally proven. Interestingly, in *P. aeruginosa*, the transport capacity of l-lysine is induced by exogenous l-arginine but not l-lysine itself [53]. Further insights into the regulation of l-lysine transport in *P. taiwanensis* VLB120 or overexpression of known lysine transporters might help to improve the capacity of l-lysine transport and thus the specific production rate of hydroxy-l-lysine. Additionally, fine-tuning of the gene expression and increasing the activity of the KDOs through enzyme engineering might further enhance the performance of the whole-cell biocatalysts.

Apart from optimizing lysine utilization and availability for the hydroxylation reaction, the Weimberg pathway is also a key for the design of an efficient *P. taiwanensis* VLB120 chassis. The extracellular accumulation of the intermediates d-xylonolactone and d-xylonate still poses a significant bottleneck, resulting in a major loss of carbon to fuel the biotransformation. However, we recently identified d-xylonolactonases and the d-xylonate transporters in *P. taiwanensis* VLB120 [31]. Increasing the expression of the respective genes may help reduce the accumulation of the intermediates and thus increase the yields on d-xylose. Our results of the biotransformations in the stirred-tank bioreactors show that every second molecule of d-xylose was used in the KDO reaction, excluding the amount of leftover intermediates at the end of the batch cultivation. It remains to be elucidated whether increasing the intracellular concentration of l-lysine by improving the capacity of l-lysine transport can also further increase the utilization of α-KG/d-xylose for the biotransformation. Alternatively, targeted modulation of α-ketoglutarate dehydrogenase, as a central enzyme in the conversion of α-KG, could help link the Fe²⁺/α-ketoglutarate-dependent oxygenase reaction to the Weimberg pathway. Since a complete knockout of the corresponding gene may impair growth, dynamic control strategies would likely be preferable to maintain viability and potentially optimize utilization of α-KG. As an alternative to additional genetic modifications, the biotransformation process might be optimized through reaction engineering. While the experiments in this study were performed as batch cultivation, a fed-batch mode with feeding of l-lysine and d-xylose might be a suitable approach to avoid growth defects due to high concentrations of l-lysine and to reduce the accumulation of the Weimberg pathway intermediates. Moreover, the use of resting cells might be suited for the process. Although the specific production rate for hydroxy-l-lysine was reduced in the second growth phase, the yield of hydroxy-l-lysine on biocatalyst (Y_Hyl/X_) was significantly higher than in the first phase. Thus, especially in combination with the optimization of l-lysine transport, resting cells could prove beneficial for the overall process. However, since the Weimberg pathway is an oxidative pathway, implementation of a cofactor regeneration system, such as NADH oxidase (NOX), might be required to ensure efficient redox balancing under resting-cell conditions [54]. Moreover, accumulation of d*-*xylonate is expected, as also observed in growing-cell biotransformations, which would affect the overall yield from d*-*xylose and might necessitate pH control during the biotransformation process. Utilization of resting cells could also enable the use of higher concentrations of Fe^2+^, which resulted in reduced growth rates in our experiments. A reduction of the growth rate was also observed with an engineered *Corynebacterium glutamicum* strain harboring a KDO from *Flavobacterium johnsoniae* for the synthesis of (4*R*)-4-hydroxy-l-lysine, when increased concentrations of Fe^2+^ were used in the cultivations [55]. In contrast, a study employing resting *E. coli* cells for whole-cell biotransformation found high concentrations of Fe^2+^ favorable for the synthesis of (3*S*)-3-hydroxy-l-lysine [49]. Nonetheless, the growing-cell biotransformation process in this study showed promising key performance metrics and presents a robust starting point for future research and optimization. With only limited strain engineering, *Pseudomonas taiwanensis* VLB120 proved very efficient for the supply of α-KG via the Weimberg pathway for Fe^2+^/α-ketoglutarate-dependent oxygenase-based biocatalysis. In other studies, concentrations of 43.0 g L^−1^ [48] and 32.4 g L^−1^ [7] were reached for the synthesis of (4*R*)-4-hydroxy-l-lysine, employing resting *E. coli* cells or immobilized enzymes, respectively. For the synthesis of (3*S*)-3-hydroxy-l-lysine final titers of up to 110.5 g L^−1^ were achieved utilizing resting *E. coli* cells with optimized l-lysine import in fed-batch mode, demonstrating the potential of further bioprocess intensification [49]. However, α-KG was used as cosubstrate in all of the mentioned studies, resulting in considerably higher costs compared to the use of d-xylose (Supplementary Table S8). Adaptations of the chassis strain and the introduction of genes coding for other Fe^2+^/α-ketoglutarate-dependent oxygenases could expand the use to other substrates/products and might enable further harnessing the wide variety of chemical reactions of Fe^2+^/α-ketoglutarate-dependent oxygenases for industrial applications using d-xylose to fuel the biotransformation.

## Conclusion

In this study, we engineered *P. taiwanensis* VLB120 for the synthesis of hydroxy-l-lysine from l-lysine, utilizing Fe^2+^/α-ketoglutarate-dependent oxygenases and d-xylose as the sole carbon source, providing α-KG via the Weimberg pathway. Although the extensive lysine catabolism of pseudomonads posed a challenge in avoiding utilization of l-lysine for biomass production, we were able to eliminate the major l-lysine degradation and to enable the synthesis of 4-hydroxy-l-lysine with our engineered *P. taiwanensis* VLB120 chassis. The supply of α-KG via the Weimberg pathway proved very efficient, as roughly every second molecule of d-xylose that was converted and entered the central carbon metabolism was used in the biotransformation. Our engineered chassis enabled multi-gram scale product formation in stirred-tank bioreactors, and its key performance metrics provide a promising basis for future chassis and bioprocess optimizations. With the growing interest in the application of Fe^2+^/α-ketoglutarate-dependent oxygenases, *P. taiwanensis* VLB120 offers great potential for the establishment of a versatile platform organism for Fe^2+^/α-ketoglutarate-dependent oxygenase-based biocatalysis in sustainable bioprocesses.

## Supplementary Information

Below is the link to the electronic supplementary material.

Supplementary Material 1.

## Data Availability

Datasets generated and analyzed during this study are available upon request.
