# Supplementary material for: Engineering Pseudomonas taiwanensis VLB120 for regio- and stereospecific hydroxylation of l-lysine fueled by the Weimberg pathway
Source: Microb Cell Fact. 2026 Feb 19;25:57. doi: 10.1186/s12934-026-02931-0 (PMC12930750; doi:10.1186/s12934-026-02931-0)
Supplement: Supplementary file 1 — Supplementary Material 1. [file 12934_2026_2931_MOESM1_ESM.docx]

**Supplementary Information**

**Engineering *Pseudomonas taiwanensis* VLB120 for regio- and stereospecific hydroxylation of l-lysine fueled by the Weimberg pathway**

Philipp Nerke, Julian Handke, Georg Hubmann, and Stephan Lütz*

*Chair for Bioprocess Engineering, Department of Biochemical and Chemical Engineering, TU Dortmund University, Emil-Figge-Straße 66, 44227 Dortmund, Germany*

*Corresponding author. E-mail: stephan.luetz@tu-dortmund.de

**Table of contents**

[1. Supplementary Figures 2](#_Toc201834135)

[2. Supplementary Tables 4](#_Toc201834136)

[3. Model Description 14](#_Toc201834137)

# Supplementary Figures

##
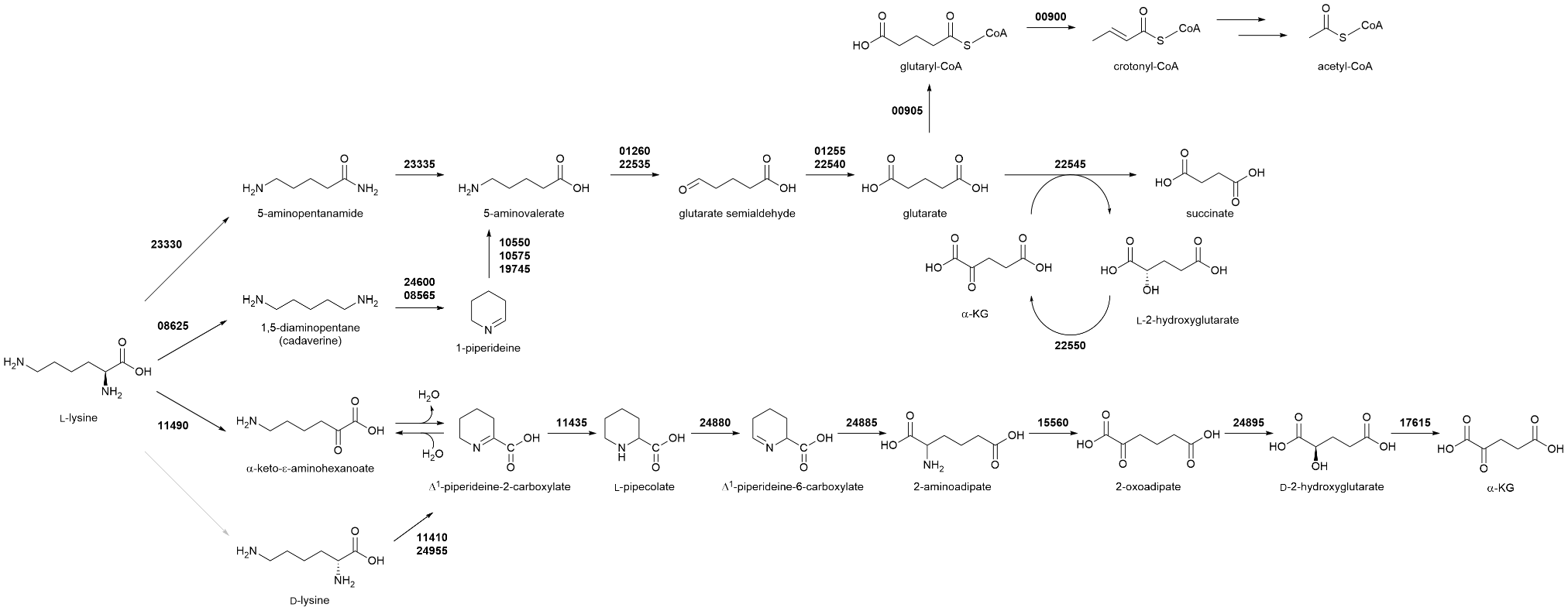


## Supplementary Figure S1: Putative l-lysine catabolic pathways in *Pseudomonas taiwanensis* VLB120. Genes were identified by Protein Blast analysis. Protein sequences corresponding to genes with known functions in l-lysine catabolism of *P. putida* KT2440 (PP) and *P. aeruginosa* PAO1 (PA) were compared against the genome of *P. taiwanensis* VLB120 (PVLB). Detected genes were PVLB_23330 (PP_0383/*davB*: 96.25 % identity), PVLB_08625 (PP_4140/*ldcC*: 93.05 % identity), PVLB_11490 (PP_3721/*aruH*: 65.80 % identity), PVLB_23335 (PP_0382/*davA*: 86.92 % identity), PVLB_01260 (PP_0214/*davT*: 96.47 % identity), PVLB_22535 (PP_0214/*davT*: 75.47 % identity), PVLB_01255 (PP_0213/*davD*: 98.12 % identity), PVLB_22540 (PP_0213/*davD*: 84.44 % identity), PVLB_00905 (PP_0159: 91.63 % identity), PVLB_00900 (PP_0158/*gcdH*: 98.73 % identity), PVLB_22545 (PP_2910/*lhgO*: 84.34 % identity), PVLB_22550 (PP_2909/*glaH*: 88.00 % identity), PVLB_24600 (PA_0299/*spuC*: 77.16 % identity), PVLB_08565 (PA_0299/*spuC*: 76.34 % identity), PVLB_10550 (PP_2801/*prr*: 79.75 % identity), PVLB_10575 (PP_2801/*prr*: 58.44 % identity), PVLB_19745 (PP_2801/*prr*: 69.83 % identity), PVLB_11410 (PP_3596/*amaD*: 87.92 % identity), PVLB_24955 (PP_4434/dadA: 79.81 % identity), PVLB_11435 (PP_3591/*dpkA*: 90.66 % identity), PVLB_24880 (PP_5257/*amaA*: 95.33 % identity), PVLB_24885 (PP_5258/*amaB*: 98.79 % identity), PVLB_15560 (PP_4108: 93.25 % identity), PVLB_24895 (PP_5260/*ydcJ*: 92.03 % identity) and PVLB_17615 (PP_4493/*ydiJ*: 95.43 % identity). For simplicity, chemical structures are depicted in uncharged form. The numbers depicted in the figure represent locus tags without PVLB prefix.


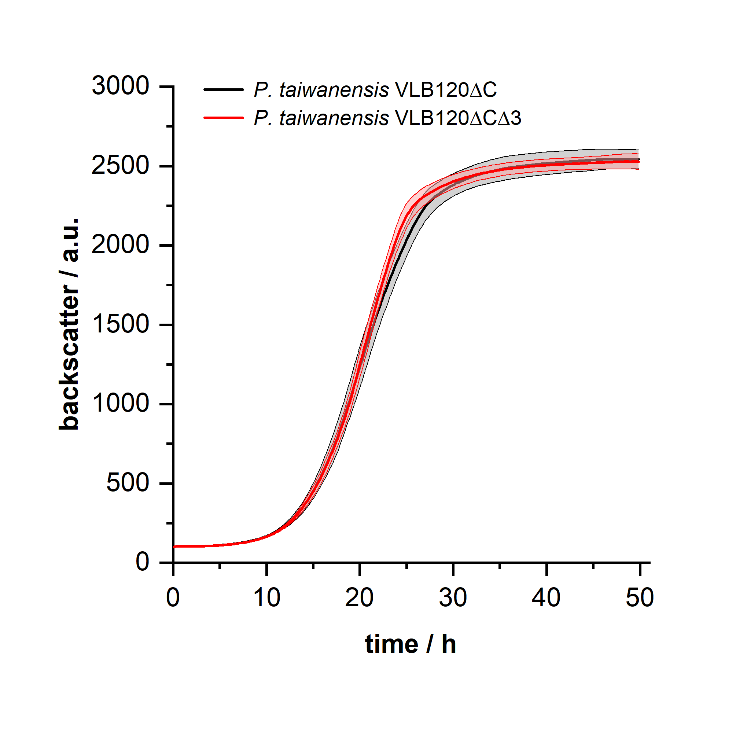
Supplementary Figure S2: Growth of *Pseudomonas taiwanensis* VLB120∆C and *P. taiwanensis* VLB120∆C∆3 on d-xylose. Cultivations were performed in M9 medium (20 g L^−1^ d-xylose and 2 g L^−1^ NH_4_Cl) at 25 mL scale, 30 °C and 200 rpm. Growth was monitored using the cell growth quantifier (CGQ) system (Aquila Biolabs). The mean values and error bands (standard deviations) are calculated from two independent biological replicates.

**
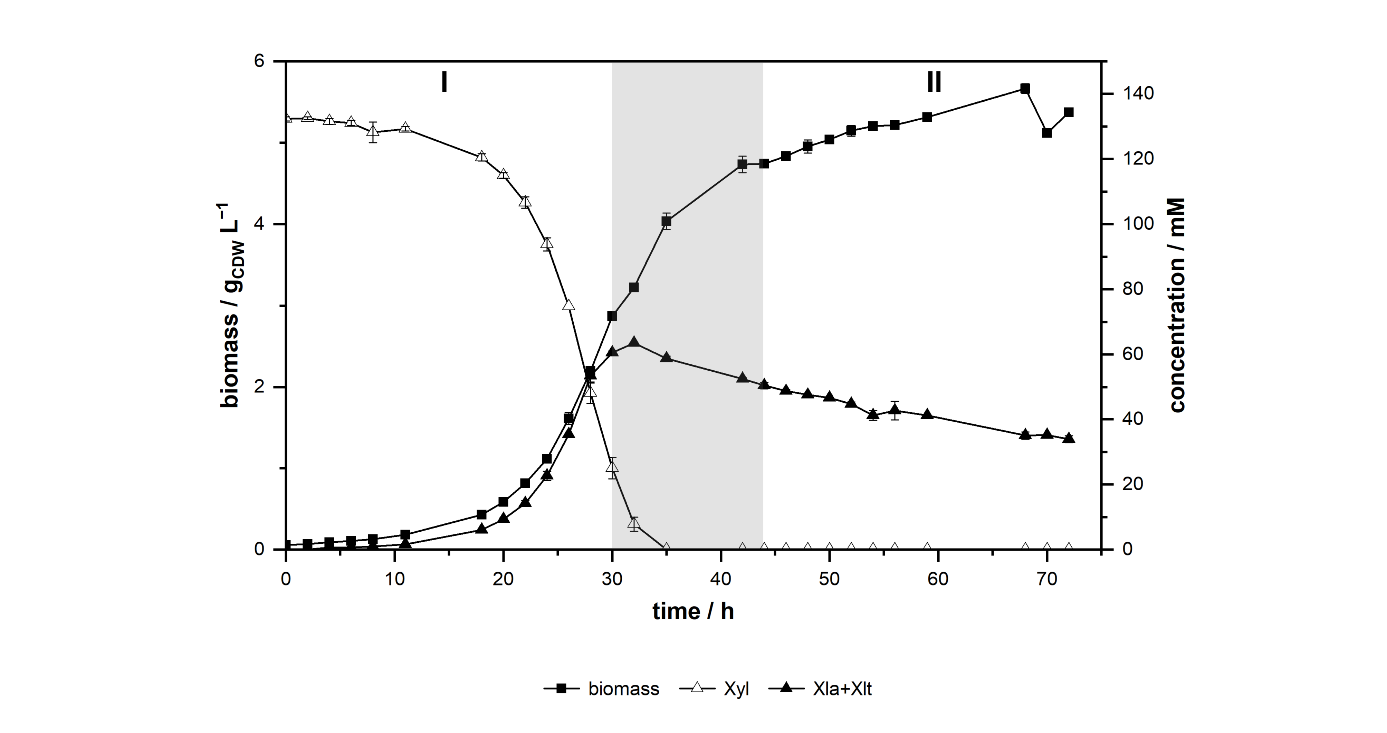
**Supplementary Figure S3: Stirred-tank bioreactor cultivation of *Pseudomonas taiwanensis* VLB120ΔCΔ3 pCom10lac_*Fspe*KDO without supplementation of l-lysine. The cultivation was performed in 200 mL M9 medium (20 g L^−1^ d‑xylose) in a stirred-tank bioreactor (DASbox, Eppendorf SE) over 72 h at 30 °C, 1,000 rpm and an aeration rate of 3 L h^−1^. The two observed growth phases are labeled I and II, and the transition phase is marked in grey. Mean values and error bars (standard deviation) are calculated from two independent biological replicates.

# Supplementary Tables

## Supplementary Table S1: Growth media and solutions.

| Media and solutions | Components | Reference |
| --- | --- | --- |
| LB-medium  (plates) | 10 g L^−1^tryptone, 5 g L^−1^ yeast extract, 10 g L^−1^ NaCl, (15 g L^−1^ agar agar) | [1] |
| M9 medium | 8.5 g L^−1^ Na_2_HPO_4_∙ H_2_O, 3 g L^−1^ KH_2_PO_4_, 0.5 g L^−1^NaCl, 1 g L^−1^ NH_4_Cl, 2 mL L^−1^ 1 M MgSO_4_, 1 mL L^−1^ US^Fe^‑trace element solution, as needed 5 g L^−1^ l-lysine or 5 g L^−1^ d-lysine, pH 7.4 | [2] |
| Modified M9 medium | 8.5 g L^−1^ Na_2_HPO_4_∙ H_2_O, 3 g L^−1^ KH_2_PO_4_, 0.5 g L^−1^NaCl, 2 g L^−1^ NH_4_Cl, 2 mL L^−1^ 1 M MgSO_4_, 1 mL L^−1^ US^Fe^‑trace element solution, 20 g L^−1^ d‑xylose, as needed l-lysine and additional FeSO_4_, pH 7.4 | [3] |
| US^Fe^‑trace element solution | 82.81 mL 37 % fuming HCL, 4.87 g L^−1^ FeSO_4_∙ 7 H_2_O, 4.12 g L^−1^ CaCl_2_∙ 2 H2O, 1.5 g L^−1^ MnCl_2_∙ 4 H_2_O, 1.87 g L^−1^ ZnSO_4_∙ 7 H_2_O, 0.2 g L^−1^ H_3_BO_3_, 0.25 g L^−1^ Na_2_MoO_4_∙ 2 H_2_O, 0.15 g L^−1^ CuCl_2_∙ 2 H_2_O, 0.4 g L^−1^ Na_2_EDTA ∙ 2 H_2_O | [4] |
| SOC-medium | 20 g L^−1^ tryptone, 5 g L^−1^ yeast extract, 0.584 g L^−1^ NaCl, 0.186 g L^−1^ KCl, 2.003 g L^−1^ MgCl_2_∙ 6 H_2_O, 10 mL L^−1^ MgSO_4_, 7.2 mL L^−1^ 50 % (w/v) glucose | [5] |
| PBS | 8 g L^−1^ NaCl, 0.2 g L^−1^ KCl, 1.44 g L^−1^ Na_2_HPO_4_, 0.24 g L^−1^ KH_2_PO_4_ | Cold Spring Harbor Protocols |

**Supplementary Table S2: Strains.** *E. coli* DH5α λpir was used for cloning and propagation of pEMG plasmids. *E. coli* DH5α was used for cloning and propagation of all other plasmids.

| Strain | Genotype / Description | Reference |
| --- | --- | --- |
| *Escherichia coli* |  |  |
| DH5α | *sup*E44 Δ*lacU*169 (φ80 *lac*ZΔM15) *hsd*R17 *rec*A1 *end*A1 *gyr*A96 *thi*-1 *rel*A1 | [5] |
| DH5α λpir | λpir lysogen of DH5a | [6] |
| *Kineococcus* species |  |  |
| *K. radiotolerans* | wild-type strain | DSM No. 14245 |
| *K. rhizosphaerae* | wild-type strain | DSM No. 19711 |
| *Pseudomonas taiwanensis* |  |  |
| VLB120ΔC | VLB120 with disruption of *styC* on the megaplasmid pSTY by insertion of a streptomycin resistance cassette (Sm^R^) | [7] |
| VLB120ΔCΔPVLB23330 | VLB120ΔC with deletion of PVLB_23330 | This work |
| VLB120ΔCΔPVLB08625 | VLB120ΔC with deletion of PVLB_08625 | This work |
| VLB120ΔCΔPVLB11490 | VLB120ΔC with deletion of PVLB_11490 | This work |
| VLB120ΔCΔ3  (VLB120ΔCΔPVLB23330  ΔPVLB08625ΔPVLB11490) | VLB120ΔC with disruption of PVLB_23330, PVLB_08625 and PVLB_11490 | This work |
| VLB120ΔCΔ3 pCom10lac | VLB120ΔCΔ3 with pCom10lac (empty vector) | This work |
| VLB120ΔCΔ3 pCom10lac_*Cpin*KDO | VLB120ΔCΔ3 with pCom10lac_*Cpin*KDO | This work |
| VLB120ΔCΔ3 pCom10lac_*Fjoh*KDO | VLB120ΔCΔ3 with pCom10lac_*Fjoh*KDO | This work |
| VLB120ΔCΔ3 pCom10lac_*Nkor*KDO | VLB120ΔCΔ3 with pCom10lac_*Nkor*KDO | This work |
| VLB120ΔCΔ3 pCom10lac_*Fspe*KDO | VLB120ΔCΔ3 with pCom10lac_*Fspe*KDO | This work |
| VLB120ΔCΔ3 pCom10lac_*Pbra*KDO | VLB120ΔCΔ3 with pCom10lac_*Pbra*KDO | This work |
| VLB120ΔCΔ3 pCom10lac_*Plum*KDO | VLB120ΔCΔ3 with pCom10lac_*Plum*KDO | This work |
| VLB120ΔCΔ3 pCom10lac_*Bpse*KDO | VLB120ΔCΔ3 with pCom10lac_*Bpse*KDO | This work |
| VLB120ΔCΔ3 pCom10lac_*Bpla*KDO | VLB120ΔCΔ3 with pCom10lac_*Bpla*KDO | This work |
| VLB120ΔCΔ3 pCom10lac_*Caci*KDO | VLB120ΔCΔ3 with pCom10lac_*Caci*KDO | This work |
| VLB120ΔCΔ3 pCom10lac_*Krad*KDO | VLB120ΔCΔ3 with pCom10lac_*Krad*KDO | This work |
| VLB120ΔCΔ3 pCom10lac_*Krhi*KDO | VLB120ΔCΔ3 with pCom10lac_*Krhi*KDO | This work |
| VLB120ΔCΔ3 pCom10lac_*Lrub*KDO | VLB120ΔCΔ3 with pCom10lac_*Lrub*KDO | This work |

## Supplementary Table S3: Plasmids. Gene and protein sequences of the KDOs are published elsewhere [8]. For *Lrub*KDO see Supplementary Table S5.

| Plasmid | Description | Reference |
| --- | --- | --- |
| pCom10lac | ColE1 and pRO1600 ori, *lac*-regulatory system (*lac*I, P_lacUV5_), Km^R^ (empty vector) | [9] |
| pCom10lac_*Cpin*KDO | pCom10lac harboring the gene coding for the KDO from *Chitinophaga pinensis* | This work |
| pCom10lac_*Fjoh*KDO | pCom10lac harboring the gene coding for the KDO from *Flavobacterium johnsoniae* | This work |
| pCom10lac_*Nkor*KDO | pCom10lac harboring the gene coding for the KDO from *Niastella koreensis* | This work |
| pCom10lac_*Fspe*KDO | pCom10lac harboring the gene coding for the KDO from *Flavobacterium* species | This work |
| pCom10lac_*Pbra*KDO | pCom10lac harboring the gene coding for the KDO from *Polyangium brachysporum* | This work |
| pCom10lac_*Plum*KDO | with pCom10lac harboring the gene coding for the KDO from *Photorhabdus luminescens* | This work |
| pCom10lac_*Bpse*KDO | pCom10lac harboring the gene coding for the KDO from *Burkholderia pseudomallei* | This work |
| pCom10lac_*Bpla*KDO | pCom10lac harboring the gene coding for the KDO from *Burkholderia plantarii* | This work |
| pCom10lac_*Caci*KDO | pCom10lac harboring the gene coding for the KDO from *Catenulispora acidiphila* | This work |
| pCom10lac_*Krad*KDO | pCom10lac harboring the gene coding for the KDO from *Kineococcus radiotolerans* | This work |
| pCom10lac_*Krhi*KDO | pCom10lac harboring the gene coding for the KDO from *Kineococcus rhizosphaerae* | This work |
| pCom10lac_*Lrub*KDO | pCom10lac harboring the gene coding for the KDO from *Leifsonia rubra* | This work |
| pET-22b(+)_*Caci*KDO | pET-22b(+) harboring the gene coding for the KDO from *Catenulispora acidiphila* | [10] |
| pET-22b(+)_*Cpin*KDO | pET-22b(+) harboring the gene coding for the KDO from *Chitinophaga pinensis* | [10] |
| pET-22b(+)_*Fjoh*KDO | pET-22b(+) harboring the gene coding for the KDO from *Flavobacterium johnsoniae* | [10] |
| pET-22b(+)_*Nkor*KDO | pET-22b(+) harboring the gene coding for the KDO from *Niastella koreensis* | [10] |
| pET-22b(+)_*Fspe*KDO | pET-22b(+) harboring the gene coding for the KDO from *Flavobacterium species* CF136 | [10] |
| pEMG | R6K ori, *lac*Za with two flanking I-SceI sites, Km^R^ | [6] |
| pEMG_*ttgV* | pEMG harboring a ~ 1 kb EcoRI-XbaI fragment for deletion of *ttgV*, Km^R^ | [11] |
| pEMG_PVLB23330 | pEMG harboring a ~ 1 kb EcoRI-XbaI fragment for deletion of PVLB_23330, Km^R^ | This work |
| pEMG_PVLB08625 | pEMG harboring a ~ 1 kb EcoRI-XbaI fragment for deletion of PVLB_08625, Km^R^ | This work |
| pEMG_PVLB11490 | pEMG harboring a ~ 1 kb EcoRI-XbaI fragment for deletion of PVLB_11490, Km^R^ | This work |
| pSW-2 | RK2 ori, *xylS*, *Pm* promoter, I-SceI gene, Gm^R^ | [6] |

Supplementary Table S4: Primer. Abbreviations: FW (forward), RV (reverse), HR (homology region).

| Primer | Sequence (5‘ → 3‘) | Description |
| --- | --- | --- |
| PPN005 | atttcacacaggatctaggaaccagtactggagaattccatATGAAGAACCTGTCTGCGTATGAAGTG | FW primer for amplification of *Caci*KDO for cloning into pCom10lac |
| PPN002 | agcttggctgcaggtcgacggatcccgggcgcgccaagcaTTAGCTGAACCTCGCAGAGACGACGC | RV primer for amplification of *Caci*KDO for cloning into pCom10lac |
| PPN006 | atttcacacaggatctaggaaccagtactggagaattccatATGAGACCCTTAGACGTGACACC | FW primer for amplification of *Cpin*KDO for cloning into pCom10lac |
| PPN004 | agcttggctgcaggtcgacggatcccgggcgcgccaagcaTTAAAGGTTTGCCAGGTGAGCGCTATATACTTTTCC | RV primer for amplification of *Cpin*KDO for cloning into pCom10lac |
| PPN007 | taggaaccagtactggagaattccatATGAAATCACAATCATTAATTGAAGATGAGA | FW primer for amplification of *Fjoh*KDO for cloning into pCom10lac |
| PPN008 | cgacggatcccgggcgcgccaagcaTTAAGCCTGATCAAAAACTTTTCCTAAATGTTC | RV primer for amplification of *Fjoh*KDO for cloning into pCom10lac |
| PPN196 | ccagtactggagaattccatATGGAAACCATCATCGAATCTCGTCAGC | FW primer for amplification of *Nkor*KDO for cloning into pCom10lac |
| PPN197 | cgggcgcgccaagcaTTACTGCTGAGAGTGGAACAGTTTACC | RV primer for amplification of *Nkor*KDO for cloning into pCom10lac |
| PPN198 | ccagtactggagaattccatATGAAATCTCAGTCTATCATGTCTGTTGAACGT | FW primer for amplification of *Fspe*KDO for cloning into pCom10lac |
| PPN199 | cgggcgcgccaagcaTTAGTCCAGGTCGAAGATTTTACCCA | RV primer for amplification of *Fspe*KDO for cloning into pCom10lac |
| PPN200 | ccagtactggagaattcCATATGCGCGCCGCAC | FW primer for amplification of *Lrub*KDO for cloning into pCom10lac |
| PPN201 | cgggcgcgccaagcaTTAGCTGTGAATCGCCGCTAAC | RV primer for amplification of *Lrub*KDO for cloning into pCom10lac |
| PPN063 | taggaaccagtactggagaattccatATGTCCTCGCTGTTCCTCG | FW primer for amplification of *Krad*KDO for cloning into pCom10lac |
| PPN064 | cgacggatcccgggcgcgccaagcaTCAGCTGAAGCTGGCCTG | RV primer for amplification of *Krad*KDO for cloning into pCom10lac |
| PPN065 | taggaaccagtactggagaattccatATGTCCTCGGTGTTCCTCG | FW primer for amplification of *Krhi*KDO for cloning into pCom10lac |
| PPN066 | cgacggatcccgggcgcgccaagcaTCAGGAGAAGCTGGCCTGC | RV primer for amplification of *Krhi*KDO for cloning into pCom10lac |
| PPN016 | CCGAATTCGTGGTCTGCAGGCCTATG | Gene knockout of PVLB_23330: FW primer of HR1 |
| PPN017 | GCATGGCGTCTCCTGGTAAAATGACCTTGCCAGAGGGC | Gene knockout of PVLB_23330: RV primer of HR1 with 5' overhang for HR2 |
| PPN018 | GCCCTCTGGCAAGGTCATTTTACCAGGAGACGCCATGC | Gene knockout of PVLB_23330: FW primer of HR2 with 5' overhang for HR1 |
| PPN019 | GCTCTAGACATGTTCGCCGTGGGTAC | Gene knockout of PVLB_23330: RV primer of HR2 |
| PPN049 | AGTATAGGGATAACAGGGTAATCTGAATTCCGCTGATATCTCCCAGCACTG | Gene knockout of PVLB_08625: FW primer of HR1 |
| PPN050 | TCGCATTCCTTGACAGCTACTACCCCCTTTCGAAC | Gene knockout of PVLB_08625: RV primer of HR1 with 5' overhang for HR2 |
| PPN051 | GGGTAGTAGCTGTCAAGGAATGCGAATGATCTCGACGC | Gene knockout of PVLB_08625: FW primer of HR2 with 5' overhang for HR1 |
| PPN052 | AAGCTTGCATGCCTGCAGGTCGACTCTAGATGTACAGCGGCAAGTCAGC | Gene knockout of PVLB_08625: RV primer of HR2 |
| PPN053 | AGTATAGGGATAACAGGGTAATCTGAATTCCGCCTCCATCTCGGCC | Gene knockout of PVLB_11490: FW primer of HR1 |
| PPN054 | GTTGCTGGGCTAGGACTGTCTCCCTGTCTGCC | Gene knockout of PVLB_11490: RV primer of HR1 with 5' overhang for HR2 |
| PPN055 | AGGGAGACAGTCCTAGCCCAGCAACCCGG | Gene knockout of PVLB_11490: FW primer of HR2 with 5' overhang for HR1 |
| PPN056 | AAGCTTGCATGCCTGCAGGTCGACTCTAGACGTCTGGCTTTACCTGTTGCTGGTATTGTGG | Gene knockout of PVLB_11490: RV primer of HR2 |
| SPPN001 | CGGTCGATCATTCAGCCCG | FW primer for sequencing/colony PCR of pCom10lac insert |
| SPPN002 | TGCCGCCAGGCAAATTCTG | RV primer for sequencing/colony PCR of pCom10lac insert |
| SPPN027 | CAGGCTGCGCAACTGTTG | FW primer for sequencing/colony PCR of pEMG insert |
| SPPN028 | GCGTTGGCCGATTCATTAATGC | RV primer for sequencing/colony PCR of pEMG insert |

## **Supplementary Table S5: Gene and protein sequence of the KDO from *Leifsonia rubra* (*Lrub*KDO).**

| Gene sequence | ATGCGCGCCGCACGAAGCAAGGAGAGCGACATTACCGTTCACGAGCTCCAATCGAGCCTTTTCACTGTTGACTCAGCCACTGCCGAAGCAATTCGTGCTGCTGCCGAGCGCATTACTGCGCATCCGAATGAGAGCCCGGATGACTTCGGTCGCCAGGCTCTCGCTGCGGCGTTTAGCTTGCCCGAAGAGGTTCGTGCCGCTGTTCTGAATTTTGCCGAGGTTGGCTCTGAGGCGGGAATCATGGTGGTGCGCGGACTCTACGTCGATGAAGACCTCACAGATACCCCGCTCAACAACAAGAGTGGACTGGGGGCTAGCACCGTATTTGCTAAAGAGATGGGCATGTTGGCTCACTTGCTCGGAAGCATGGTCGCTTACGAAGCTGAGGGCAACGGTCACCTCATCCAAGACATGGTCCCCAACCCGAAGCTTGCGGTAACGCAGCAGTCGCAGGGTTCAAAGGTGGAGCTTGAAGCCCACACCGAGCAGTGCTTCTCGGTATTCAAGCCCGATTACGTGATTCTTGGAGCCTTGCGCGGCGACGAGAACGCAAAGACCTATGCTTTCTCGGGCCGCAAGCTCGTTCAGCACCTTTCCCCCGAAGAGGTCGCTAAGCTGCGCCAGCCGCTGTGGGCCACGACGATCGATGAGTCTTTCCAGGCATACATCCCGCGCCCGGATGACGTGCGTGGCCCCTTCCCGATCCTCACCGGACCGGAAGACGACCCGTATATCTTGGTCGATCAAGACCTCATGCACGGCATCACCGCTGAAGCGCAGCGACTGCTGGGTAAGGTGGTTGAGACCTACATCGAGCACCGGGATGCTCACGTGCTGCAGCCGGGTGACTTGCTGATGCTCGACAACCTGCGCGCGATGCATGGTCGCTCAATGTATGCGCCCCGCTTCGATGGCAAGGATCGTTTTATTGCTCGCGGTTTCGTTGTGCGCGACCGTCGCAAGCTCTGGCCACAGCTGCTCGGAGACCGCCGCACGTTAGCGGCGATTCACAGCTAA |
| --- | --- |
| Protein sequence | MRAARSKESDITVHELQSSLFTVDSATAEAIRAAAERITAHPNESPDDFGRQALAAAFSLPEEVRAAVLNFAEVGSEAGIMVVRGLYVDEDLTDTPLNNKSGLGASTVFAKEMGMLAHLLGSMVAYEAEGNGHLIQDMVPNPKLAVTQQSQGSKVELEAHTEQCFSVFKPDYVILGALRGDENAKTYAFSGRKLVQHLSPEEVAKLRQPLWATTIDESFQAYIPRPDDVRGPFPILTGPEDDPYILVDQDLMHGITAEAQRLLGKVVETYIEHRDAHVLQPGDLLMLDNLRAMHGRSMYAPRFDGKDRFIARGFVVRDRRKLWPQLLGDRRTLAAIHS* |

Supplementary Table S6: Stirred-tank bioreactor cultivation parameters with added l-lysine. Colored values (0 to 30 h and 44 to 59 h) represent data used for model generation and analysis with gProms. Measured parameters were biomass concentration, concentration of d-xylose (Xyl), combined concentration of d-xylonolactone and d-xylonate (XlaXlt), concentration of l-lysine (Lys) and concentration of hydroxy-l-lysine (Hyl). n.d. – not detected, ex. – value excluded.

| Replicate 1 | | | | | |
| --- | --- | --- | --- | --- | --- |
| time / h | biomass / g_CDW_ L^−1^ | Xyl / mM | XlaXlt / mM | Lys / mM | Hyl / mM |
| 0 | 0.050 | 130.7 | 0.0 | 92.1 | 0.0 |
| 2 | 0.075 | 130.4 | 0.0 | 86.6 | 0.0 |
| 4 | 0.108 | 130.0 | 0.0 | 85.8 | 0.0 |
| 6 | 0.129 | 129.3 | 0.2 | 91.4 | 0.0 |
| 8 | 0.145 | 128.4 | 0.3 | 90.9 | 0.0 |
| 11 | 0.203 | 126.0 | 0.9 | 90.7 | 0.0 |
| 18 | 0.641 | 106.5 | 13.2 | 86.0 | 1.6 |
| 20 | 0.900 | 98.1 | 18.9 | 86.0 | 2.3 |
| 22 | 1.233 | 85.0 | 26.9 | 84.9 | 2.8 |
| 24 | 1.598 | 68.1 | 37.8 | 81.9 | 3.7 |
| 26 | 2.105 | ex. | ex. | ex. | ex. |
| 28 | 2.751 | 29.8 | 53.8 | 81.6 | 7.3 |
| 30 | 3.253 | 15.0 | 54.5 | 76.8 | 9.8 |
| 32 | 3.371 | 5.1 | 54.0 | 78.4 | 13.7 |
| 35 | 4.057 | n.d. | 51.7 | 70.1 | 18.0 |
| 42 | 4.416 | n.d. | 43.5 | 58.3 | 30.4 |
| 44 | 4.487 | n.d. | 41.2 | 55.0 | 34.2 |
| 46 | 4.569 | n.d. | 39.2 | 52.6 | 38.0 |
| 48 | 4.610 | n.d. | 36.7 | 48.0 | 39.2 |
| 50 | 4.631 | n.d. | 35.2 | 44.3 | 40.0 |
| 52 | 4.784 | n.d. | 33.8 | 42.5 | 42.7 |
| 54 | 4.846 | n.d. | 32.3 | 42.3 | 46.5 |
| 56 | 4.877 | n.d. | 31.1 | 41.2 | 48.9 |
| 59 | 4.948 | n.d. | 29.9 | 40.8 | 51.8 |
| 68 | 4.825 | n.d. | 24.9 | 33.0 | 54.8 |
| 70 | 4.825 | n.d. | 24.1 | 33.2 | 56.7 |
| 72 | 5.020 | n.d. | 23.5 | 29.0 | 52.3 |

Supplementary Table S6 (continued): Stirred-tank bioreactor cultivation parameters with added l-lysine.

| Replicate 2 | | | | | |
| --- | --- | --- | --- | --- | --- |
| time / h | biomass / g_CDW_ L^−1^ | Xyl / mM | XlaXlt / mM | Lys / mM | Hyl / mM |
| 0 | 0.053 | 131.0 | 0.0 | 89.4 | 0.0 |
| 2 | 0.076 | 130.8 | 0.0 | 89.4 | 0.0 |
| 4 | 0.111 | 129.8 | 0.0 | 89.2 | 0.0 |
| 6 | 0.128 | 129.1 | 0.2 | 89.8 | 0.0 |
| 8 | 0.144 | 128.6 | 0.4 | 86.9 | 0.0 |
| 11 | 0.203 | 126.1 | 0.9 | 87.5 | 0.0 |
| 18 | 0.621 | 107.7 | 12.6 | 89.2 | 1.7 |
| 20 | 0.877 | 99.5 | 18.2 | 84.5 | 2.3 |
| 22 | 1.164 | 87.1 | 25.6 | 84.3 | 2.7 |
| 24 | 1.532 | 71.2 | 35.5 | 81.6 | 3.5 |
| 26 | 2.034 | 50.2 | 47.5 | 82.3 | 5.2 |
| 28 | 2.536 | 31.4 | 54.2 | 82.0 | 7.3 |
| 30 | 3.084 | 16.1 | 54.6 | 81.8 | 10.0 |
| 32 | 3.299 | 6.2 | 54.6 | 76.4 | 12.4 |
| 35 | 4.067 | n.d. | 51.9 | 72.0 | 17.3 |
| 42 | 4.323 | n.d. | 43.9 | 61.3 | 30.8 |
| 44 | 4.354 | n.d. | 42.0 | 51.6 | 30.7 |
| 46 | 4.364 | n.d. | 39.8 | 55.8 | 38.4 |
| 48 | 4.661 | n.d. | 37.3 | 49.0 | 38.1 |
| 50 | 4.559 | n.d. | 35.8 | 50.1 | 43.9 |
| 52 | 4.661 | n.d. | 34.3 | 47.4 | 45.9 |
| 54 | 4.702 | n.d. | 32.5 | 43.5 | 46.5 |
| 56 | 4.713 | n.d. | 31.5 | 42.7 | 49.1 |
| 59 | 4.877 | n.d. | 30.3 | 39.0 | 47.9 |
| 68 | 5.010 | n.d. | 25.2 | 34.6 | 55.8 |
| 70 | 4.631 | n.d. | 24.5 | 33.4 | 56.0 |
| 72 | 4.989 | n.d. | 23.9 | 31.6 | 55.2 |

Supplementary Table S7: Stirred-tank bioreactor cultivation parameters without supplementation of l-lysine. Colored values (0 to 30 h and 44 to 59 h) represent data used for model generation and analysis with gProms. Measured parameters were biomass concentration, concentration of d-xylose (Xyl) and combined concentration of d-xylonolactone and d-xylonate (XlaXlt). n.d. – not detected, ex. – value excluded.

|  |  | Replicate 1 | | |  | Replicate 2 | | |
| --- | --- | --- | --- | --- | --- | --- | --- | --- |
| time / h |  | biomass / g_CDW_ L^−1^ | Xyl / mM | XlaXlt / mM |  | biomass / g_CDW_ L^−1^ | Xyl / mM | XlaXlt / mM |
| 0 |  | 0.052 | 132.8 | 0.0 |  | 0.054 | 131.9 | 0.0 |
| 2 |  | 0.070 | 132.9 | 0.0 |  | 0.069 | 132.2 | 0.0 |
| 4 |  | 0.088 | 132.2 | 0.6 |  | 0.087 | 130.9 | 0.6 |
| 6 |  | 0.105 | 131.6 | 0.6 |  | 0.108 | 130.5 | 0.7 |
| 8 |  | 0.124 | 125.9 | 0.9 |  | 0.134 | 130.5 | 1.0 |
| 11 |  | 0.175 | 129.8 | 1.5 |  | 0.191 | 128.8 | 1.6 |
| 18 |  | 0.421 | 121.3 | 5.9 |  | 0.433 | 119.7 | 6.1 |
| 20 |  | 0.566 | 115.6 | 9.0 |  | 0.602 | 114.4 | 9.6 |
| 22 |  | 0.799 | 107.9 | 13.6 |  | 0.828 | 105.4 | 14.8 |
| 24 |  | 1.091 | 95.2 | 21.6 |  | 1.137 | 92.3 | 23.6 |
| 26 |  | 1.557 | ex. | ex. |  | 1.665 | 74.7 | 35.4 |
| 28 |  | 2.182 | 50.3 | 52.1 |  | 2.213 | 45.8 | 54.8 |
| 30 |  | 2.833 | 27.3 | 60.2 |  | 2.910 | 22.7 | 60.9 |
| 32 |  | 3.237 | 9.3 | 63.4 |  | 3.207 | 6.3 | 63.6 |
| 35 |  | 4.108 | n.d. | 59.3 |  | 3.965 | n.d. | 58.1 |
| 42 |  | 4.805 | n.d. | 52.8 |  | 4.661 | n.d. | 52.3 |
| 44 |  | 4.743 | n.d. | 51.2 |  | 4.743 | n.d. | 49.8 |
| 46 |  | 4.805 | n.d. | 49.0 |  | 4.866 | n.d. | 48.3 |
| 48 |  | 4.897 | n.d. | 47.7 |  | 5.010 | n.d. | 47.4 |
| 50 |  | 5.020 | n.d. | 46.6 |  | 5.061 | n.d. | 46.5 |
| 52 |  | 5.102 | n.d. | 44.1 |  | 5.194 | n.d. | 45.1 |
| 54 |  | 5.184 | n.d. | 40.2 |  | 5.225 | n.d. | 42.2 |
| 56 |  | 5.204 | n.d. | 44.6 |  | 5.225 | n.d. | 40.6 |
| 59 |  | 5.317 | n.d. | 41.2 |  | 5.307 | n.d. | 41.0 |
| 68 |  | 5.625 | n.d. | 34.2 |  | 5.706 | n.d. | 35.8 |
| 70 |  | 5.123 | n.d. | 35.3 |  | 5.112 | n.d. | 35.0 |
| 72 |  | 5.338 | n.d. | 34.7 |  | 5.409 | n.d. | 33.2 |

Supplementary Table S8: Costs of α-ketoglutarate and d-xylose.

|  | α-ketoglutarate | d-xylose |
| --- | --- | --- |
| Vendor | Sigma-Aldrich^1^ | Sigma-Aldrich^2^ |
| Quantity | 10 kg | 10 kg |
| Purity | > 99 % | > 99 % |
| Price | 3,980 € | 372.00 € |
| Price per kg | 398 € | 37.20 € |

1. https://www.sigmaaldrich.com/DE/de/product/mm/105194 (accessed 25 November 2025)

2. https://www.sigmaaldrich.com/DE/de/product/aldrich/w360600 (accessed 25 November 2025)

# Model Description

The exponential growth model consisted of the following differential equations to determine the amount of biomass, d-xylose, l-lysine, hydroxy-l-lysine and the combined intermediates, and the model of the first growth phase was given by,

$\frac{dc_{X}}{dt}=\mu\cdot c_{X}$ (1)

$\frac{dc_{Xyl}}{dt}=- r_{Xyl}\cdot c_{X}$ (2)

$\frac{dc_{Lys}}{dt}=- \frac{1}{Y_{Hyl /Lys}}\cdot r_{Hyl}\cdot c_{X}$ (3)

$\frac{dc_{Hyl}}{dt}=r_{Hyl}\cdot c_{X}$ (4)

$\frac{dc_{XlaXlt}}{dt}=r_{XlaXlt}\cdot c_{X}$ (5)

where c_x_ is the measured biomass concentration [g_CDW_ L^−1^], c_Xyl_ the d-xylose concentration [mmol L^−1^], c_Lys_ the l-lysine concentration [mmol L^−1^], c_Hyl_ the hydroxy-l-lysine concentration [mmol L^−1^] and c_XlaXlt_ the concentration of combined d-xylonolactone and d-xylonate [mmol L^−1^]. The growth rate µ [h^−1^] was assumed to be constant in each growth phase, resulting in the constant specific rates,

$r_{Xyl}=\frac{\mu}{Y_{X/Xyl}}$ (6)

$r_{Lys}=- \frac{1}{Y_{Hyl /Lys}}\cdot r_{Hyl}$ (7)

$r_{Hyl}=Y_{Hyl/X} \cdot\mu$ (8)

$r_{XlaXlt}=Y_{XlaXlt/X} \cdot\mu$ (9)

where r_Xyl_ denotes the specific d-xylose uptake rate [mmol g_CDW_^−1^ h^−1^], r_Lys_ the specific l-lysine uptake rate [mmol g_CDW_^−1^ h^−1^], r_Hyl_ the specific hydroxy-l-lysine production rate [mmol g_CDW_^−1^ h^−1^], r_XlaXlt_ the specific combined d-xylonolactone and d-xylonate production rate [mmol g_CDW_^−1^ h^−1^]. The specific rates were calculated from the growth rate µ and the corresponding specific biomass yields, i.e., Y_X/Xyl_ biomass yield [g_CDW_ mmol^−1^], Y_Hyl/X_ hydroxy-l-lysine yield [mmol g_CDW_^−1^] and Y_XlaXlt/X_ combined d-xylonolactone and d-xylonate yield [mmol g_CDW_^−1^]. The l-lysine uptake rate was assumed to correlate with the specific hydroxy-l-lysine production rate, taking into account the conversion yield of hydroxy-l-lysine, Y_Hyl/Lys_ [mmol/mmol]. To take into account the d-xylose depletion and the consumption of the Weimberg pathway intermediates d-xylonolactone and d-xylonate, the second growth phase model omits the equations (2) and (6) and instead accounts for the consumption of the combined intermediates given by:

$\frac{dc_{XlaXlt}}{dt}=- r_{XlaXlt}\cdot c_{X}$ (10)

$r_{XlaXlt}=\frac{\mu}{Y_{X/XlaXlt}}$ (11)

where r_XlaXlt_ denotes the specific combined d-xylonolactone and d-xylonate uptake rate [mmol g_CDW_^−1^ h^−1^] and Y_X/XlaXlt_ the biomass yield [mmol g_CDW_^−1^] of the second growth phase.

In experiments without l-lysine we excluded the equation of l-lysine (3) and hydroxy-l-lysine (4). The parameters were estimated based on two replicate cultivations, where the initial concentrations were estimated for each replicate. The specific rates r_Xyl_, r_Hyl_ and r_XlaXlt_ were calculated from the estimated parameters, i.e. the growth rate and corresponding yields, as indicated in the equations (6) – (9) and (11). To quantify the specific rates’ uncertainties, we calculated the standard error of the growth rate and corresponding yields, assuming that all parameter values are distributed normally, and that the covariance of the parameters was insignificant. The standard errors of the derived parameters were determined using the appropriate error propagation.

## Cited literature

1. Bertani G. Studies on lysogenesis. I. The mode of phage liberation by lysogenic *Escherichia coli*. J Bacteriol. 1951;62:293–300.

2. Sambrook J, Russell DW. Molecular cloning: a laboratory manual. 3. Cold Spring Harbor, NY: Cold Spring Harbor Laboratory Press; 2001.

3. Nerke P, Korb J, Haala F, Hubmann G, Lütz S. Metabolic bottlenecks of *Pseudomonas taiwanensis* VLB120 during growth on D-xylose via the Weimberg pathway. Metab Eng Commun. 2024;18:e00241.

4. Bühler B, Bollhalder I, Hauer B, Witholt B, Schmid A. Use of the two-liquid phase concept to exploit kinetically controlled multistep biocatalysis. Biotechnol Bioeng. 2003;81:683–94.

5. Hanahan D. Studies on transformation of *Escherichia coli* with plasmids. J Mol Biol. 1983;166:557–80.

6. Martínez-García E, de Lorenzo V. Engineering multiple genomic deletions in Gram-negative bacteria: Analysis of the multi-resistant antibiotic profile of *Pseudomonas putida* KT2440. Environ Microbiol. 2011;13:2702–16.

7. Park J-B, Bühler B, Panke S, Witholt B, Schmid A. Carbon metabolism and product inhibition determine the epoxidation efficiency of solvent-tolerant *Pseudomonas* sp. strain VLB120ΔC. Biotechnol Bioeng. 2007;98:1219–29.

8. Rolf J, Nerke P, Britner A, Krick S, Lütz S, Rosenthal K. From cell-free protein synthesis to whole-cell biotransformation: Screening and identification of novel α-ketoglutarate-dependent dioxygenases for preparative-scale synthesis of hydroxy-L-lysine. Catalysts. 2021;11.

9. Lindmeyer M. *Pseudomonas* and heterogeneity – benefits and challenges for strain and process engineering. Aachen: Shaker Verlag; 2016.

10. Baud D, Saaidi P, Monfleur A, Harari M, Cuccaro J, Fossey A, et al. Synthesis of Mono‐ and Dihydroxylated Amino Acids with New α‐Ketoglutarate‐Dependent Dioxygenases: Biocatalytic Oxidation of C-H Bonds. ChemCatChem. 2014;6:3012–7.

11. Volmer J, Neumann C, Bühler B, Schmid A. Engineering of *Pseudomonas taiwanensis* VLB120 for constitutive solvent tolerance and increased specific styrene epoxidation activity. Appl Environ Microbiol. 2014;80:6539–48.
